# Deciphering the Interplay Among Inflammatory Bowel Disease, Gut Microbiota, and Inflammatory Biomarkers in the Risk of Colorectal Cancer

**DOI:** 10.1155/mi/4967641

**Published:** 2025-03-08

**Authors:** Chenyu Luo, Bowen Tian, Yueyang Zhou, Jiahui Luo, Qing Shang, Si Yu, Min Dai, Yue Li, Hongda Chen

**Affiliations:** ^1^Center for Prevention and Early Intervention, National Infrastructures for Translational Medicine, Institute of Clinical Medicine, Peking Union Medical College Hospital, Chinese Academy of Medical Science and Peking Union Medical College, Beijing 100730, China; ^2^Department of Cancer Epidemiology, National Cancer Center/National Clinical Research Center for Cancer/Cancer Hospital, Chinese Academy of Medical Sciences and Peking Union Medical College, Beijing 100021, China; ^3^Department of Gastroenterology, Peking Union Medical College Hospital, Chinese Academy of Medical Science and Peking Union Medical College, Beijing 100730, China

**Keywords:** colorectal cancer, gut microbiota, inflammatory biomarkers, inflammatory bowel disease

## Abstract

**Background:** Patients with inflammatory bowel disease (IBD) have an elevated colorectal cancer (CRC) risk, though the etiology remains unclear. This study aimed to elucidate the interplay among IBD, gut microbiota (GM), inflammatory biomarkers, and CRC risk.

**Methods:** First, we employed cohort analysis using the UK Biobank (UKB), linkage disequilibrium score regression (LDSC), and Mendelian randomization (MR) analyses to investigate the association between IBD and CRC. Second, inflammatory biomarkers' indirect effect was assessed using mediation analysis. Third, the causal effects of IBD on GM and GM on inflammatory biomarkers were evaluated using MR. Finally, we constructed a disease severity biomarker score and evaluated its CRC risk stratification performance.

**Results:** Among 441,321 participants, IBD was associated with a 1.78-fold (95% confidence interval (CI): 1.45–2.18) increased risk of CRC. While LDSC and MR analyses showed no genetic correlation between IBD and CRC, mediation analyses revealed that C-reactive protein (CRP) and neutrophil-to-lymphocyte ratio (NLR) significantly mediated 10.41% and 9.97% of the IBD–CRC association, respectively. IBD increased the GM abundance of *Rikenellaceae RC9 gut group*, and decreased *Lactobacillaceae* and *Ruminococcus 2*, which in turn affected CRP, neutrophils, and lymphocytes. Notably, IBD decreased the abundance of *Ruminococcus 2* after Bonferroni correction (*β* = −9.463, *p*=0.0002). A disease severity biomarker score comprising of CRP, platelets, platelet-to-lymphocyte ratio (PLR), NLR, hemoglobin (Hgb), and albumin was constructed. IBD patients with the highest scores had a 3.07-fold (95% CI: 1.35–7.00) higher CRC risk compared to those with the lowest scores.

**Conclusions:** IBD alters the microbial abundance of *Rikenellaceae RC9 gut group*, *Lactobacillaceae*, and *Ruminococcus 2*, thereby, influencing inflammatory biomarkers including CRP, neutrophils, and lymphocytes, which mediate the increased risk of CRC in IBD patients. The constructed biomarker score enables individualized CRC risk stratification in IBD patients.

## 1. Introduction

Inflammatory bowel disease (IBD), comprising the two major subtypes Crohn's disease (CD) and ulcerative colitis (UC), are chronic, relapsing inflammatory disorders of the gastrointestinal tract. IBD patients have a significantly higher risk of developing colorectal cancer (CRC), which is closely associated with the “inflammation–dysplasia–carcinoma” pathophysiological sequence [1]. However, the exact pathogenesis of IBD-associated CRC remains unclear, it may occurs as a result of complicated interactions between genetic predisposition, environmental factors, inflammatory processes, gut microbiota (GM) dysbiosis, et cetera [2].

The GM has diverse and essential roles in host metabolism, immune processes, and inflammatory responses. GM have been implicated in the pathophysiology of inflammation, especially in patients with confirmed IBD [3]. Perturbations of the GM, termed GM dysbiosis, are commonly observed in IBD. It involves the abnormal accumulation or increased virulence of certain resident bacterial populations, thereby, transforming former symbionts into colonizing “pathogenic” bacteria that can trigger intestinal inflammation [4]. GM dysbiosis may be one of the potential mechanisms contributing to the sustained chronic inflammation in IBD patients.

Concurrently, chronic persistent inflammation can also lead to abnormalities in a series of biomarkers, such as decreased serum albumin levels and elevated C-reactive protein (CRP) [5]. These biomarkers can effectively reflect the disease severity of IBD, and there is promise in utilizing them to predict the risk of IBD-associated CRC [6]. GM dysbiosis and related biomarker abnormalities may be key links in the progression from IBD to CRC; however, relevant studies unraveling this connection are lacking.

**Figure 1:**
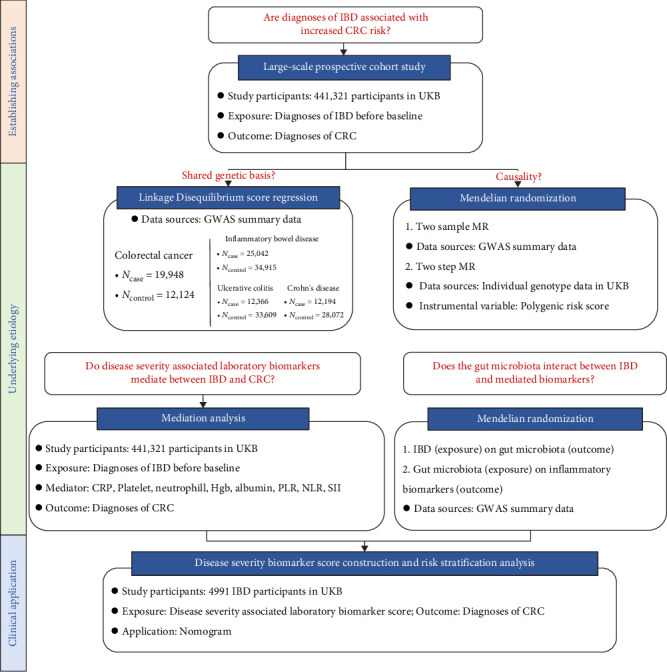
Overview of the workflow, aiming at investigating the association between IBD and CRC and elucidating the underlying IBD–GM–inflammation–CRC pathway. CRC, colorectal cancer; CRP, C-reactive protein; GM, gut microbiota; GWAS, genome-wide association analysis; Hgb, hemoglobin; IBD, inflammatory bowel disease; MR, mendelian randomization; NLR, neutrophil-to-lymphocyte ratio; PLR, platelet-to-lymphocyte ratio; SII, systemic immune-inflammation index; UKB, UK Biobank.

Our study aimed to elucidate the specific interplays among IBD, GM, IBD severity-associated biomarkers, and CRC. We hypothesize that IBD-induced changes in GM may promote CRC development through inflammatory biomarkers, suggesting the existence of an IBD–GM–inflammation–CRC pathway. We conducted several studies (Figure 1 and Table 1) to prove our hypothesis.

## 2. Methods

Summary of the research questions and data sources used, as well as key assumptions, strengths, and limitations of each method applied in the present study are presented in Table 1.

### 2.1. Study 1: Assessment of the Association Between IBD and CRC in a Population-Based Cohort

#### 2.1.1. Study Population

Study 1 was conducted using the large-scale prospective UK Biobank (UKB) cohort. Briefly, UKB recruited over 500,000 individuals aged from 40 to 69 years between 2006 and 2010. Details of the UKB study can be found elsewhere [7]. Ethical approval for the UKB was obtained from the North West-Haydock Research Ethics Committee (REC reference: 16/NW/0274), and all participants provided written informed consent.

We excluded participants who had been diagnosed with any invasive cancer at baseline, withdrew consent, and lacked genetic data (Figure S1).

#### 2.1.2. Assessment of IBD and CRC Diagnosis

Participants diagnosed with IBD were identified utilizing International Classification of Diseases, 10th revision (ICD-10) codes (K51 for UC and K50 for CD). The IBD group consisted of participants diagnosed with IBD before enrollment. The non-IBD group included others without IBD diagnosis at baseline, irrespective of subsequent IBD development during follow-up. Participants with a dual diagnosis of both UC and CD were classified according to the most recent ICD-10 code. CRC cases was identified using ICD-10 (C18–C20). Complete follow-up was available through November 30, 2022.

#### 2.1.3. Assessment of Covariates

Given that multiple lifestyle-related factors have been associated with an increased risk of CRC and often cluster together, we applied a modified healthy lifestyle index (HLI) based on the World Cancer Research Fund/American Institute of Cancer Research (WCRF/AICR) Cancer Prevention Recommendations [8, 9]. Detailed definition can be found in Table S1. In addition, we considered covariates including age, sex, ethnicity, assessment center, education level, Townsend deprivation index, CRC screening history, family history of CRC, standard polygenic risk score (PRS) for CRC, and IBD disease course. Missing values in continuous covariates were imputed using the sex-specific mean, while those in categorical covariates were assigned to “unknown.”

#### 2.1.4. Statistical Analysis

Participants were followed from recruitment until CRC diagnosis, death, loss to follow-up, or the study's end, whichever occurred first. Schoenfeld residuals–based tests confirmed that the proportional hazards assumption held, allowing the use of Cox proportional hazards models. Hazard ratios (HRs) with 95% confidence intervals (CIs) were reported. Two multivariable-adjusted models were applied: Model 1 adjusted for age, sex, and race, while Model 2 included additional adjustments for the Townsend deprivation index, assessment center, educational level, CRC screening history, family history of CRC, standard PRS for CRC, HLI, and IBD disease course.

Subgroup analyses were performed according to IBD subtype and sex. Sensitivity analyses were performed after: (1) excluding CRC cases within the first 2 years of follow-up; (2) excluding participants who had IBD diagnosis after recruitment; (3) excluding IBD patients who had dual diagnosis of both UC and CD. Analyses were performed using R version 4.1.2. *p* < 0.05 indicated a significant difference.

### 2.2. Study 2: Evaluation of the Genetic Correlation and Causal Effect Between IBD and CRC

#### 2.2.1. Linkage Disequilibrium Score Regression (LDSC) Analysis

LDSC allows the estimation of the genetic correlation (*R*_g_) between various traits. We used the largest available genome-wide association study (GWAS) summary statistics on IBD [10], UC [10], CD [10], and CRC [11]. We performed LDSC analysis using pre-computed LD scores from the 1000 Genomes project European data, and restricted it to only HapMap3 single nucleotide polymorphisms (SNPs) to minimize low imputation quality bias. The LDSC software (version.1.0.1) was used [12].

#### 2.2.2. Mendelian Randomization (MR) Analysis

MR is a method used to assess causal relationships between exposures and outcomes using genetic variants as instrumental variables (IVs) [13]. To provide valid causal inference, three key assumptions must be met: the genetic variants (1) are robustly associated with the exposure (risk factor) of interest; (2) are unrelated to potential confounding variables that might influence both the exposure and the outcome; (3) affecting the outcome exclusively through the exposure pathway [9]. We performed two-sample MR analyses to assess the causal effect between IBD, UC, CD, and CRC. Variants were pruned by clumping using the 1000 Genomes Project phase 3 European data, with an *r*^2^ threshold of 0.01 within a 1000 Kb window. IVs were then identified as being robustly associated with each trait (*p* ≤ 5 × 10^−8^). The alleles were harmonized and variants identified as palindromic were removed. Details of the harmonized dataset are provided in Table S2.

The inverse-variance weighted (IVW) method was applied. We assessed the strength of IVs by estimating the mean *F* statistic and post hoc statistical power. We performed sensitivity analyses, including MR-Egger regression, weighted median, simple mode, and weighted mode. Two-sample MR analyses were performed using the “TwoSampleMR” R Package. We also performed a two-step MR analysis using standard PRS for UC and CD in UKB as IVs [14].

### 2.3. Study 3: Mediation Analysis of Disease Severity-Associated Laboratory Biomarkers on the Association Between IBD and CRC

The study population, assessment of IBD and CRC diagnosis, and evaluation of covariates were consistent with study 1.

#### 2.3.1. Assessment of Laboratory Biomarkers

Serum levels of biochemistry biomarkers were measured using a Beckman Coulter AU5800 analyzer (Beckman Coulter, UK, Ltd). The complete blood counts were measured using a Beckman Coulter LH750 system (Beckman Coulter, Brea, CA) as per manufacturer's procedures [15].

We selected biomarkers associated with IBD disease severity based on previous literature and the data available in UKB [6, 16]. These biomarkers encompassed albumin, CRP, hemoglobin (Hgb), platelets, neutrophils, lymphocytes, platelet-to-lymphocyte ratio (PLR), neutrophil-to-lymphocyte ratio (NLR), and the systemic immune-inflammation index (SII). The SII was calculated as platelet count × neutrophil count/lymphocyte count.

#### 2.3.2. Statistical Analysis

We performed multiple imputation by chained equations with predictive mean matching to impute missing biomarker values. We used marginal structural models (MSMs) for mediation analysis [17] and employed an Aalen model for the survival outcome [18].

### 2.4. Study 4: Association Between IBD, GM, and Biomarkers With Significant Mediating Effects

We performed two-sample MR analyses to assess the potential causal effect of IBD on GM and GM on the inflammatory biomarkers with significant mediating effects. The summary statistics of GM were collected from MiBioGen consortium [19]. Information is detailed in Table S3. The main methodology of MR is described in Study 2. Since a limited number of SNPs related to GM, we used *p* < 1 × 10^−5^ as the significant thresholds for selecting IVs related to GM. Cochran's *Q* statistics was employed to quantify the heterogeneity of IVs. The intercept of MR-Egger regression examined the presence of potential pleiotropy. Bonferroni correction was conducted to correct the bias of multiple testing.

### 2.5. Study 5: Construction of Disease Severity Associated Biomarker Score and Evaluation of Its use in Risk Stratification for IBD Patients

In this study, we restricted participants who were diagnosed with IBD before recruitment in UKB.

#### 2.5.1. Disease Severity-Associated Biomarker Selection and Score Construction

A circular correlation heatmap was generated and highly correlated biomarkers were excluded. Collinearity between the biomarkers was assessed through the variance inflation factor (VIF) [20].

Selected biomarkers were dichotomized according to the median value. For CRP, platelets, NLR, and PLR, we assigned 1 point to measurements above the median. In contrast, for Hgb and albumin, 1 point was assigned for measurements below the median. The assigned points for each biomarker were summed and then categorized into four groups: low risk (0–1 point), low–moderate risk (2 points), moderate–high risk (3–4 points), and high risk (5–6 points).

#### 2.5.2. Statistical Analysis

Two models were constructed as stated in study 1. Subgroup analyses were performed according to IBD subtype and sex. Sensitivity analysis was performed after excluding IBD patients who had dual diagnosis of both UC and CD. We also constructed a nomogram leveraging the identified risk factors for clinical application.

## 3. Results

### 3.1. Study 1

Of the 441,321 participants included in the analysis, 4991 (1.1%) had a diagnosis of IBD. Among IBD cases, 3301 (66.1%) had UC and 1690 (33.9%) had CD. The baseline characteristics are shown in Table 2. Compared to non-IBD participants, those with IBD were older and more likely to be male.

**Figure 2:**
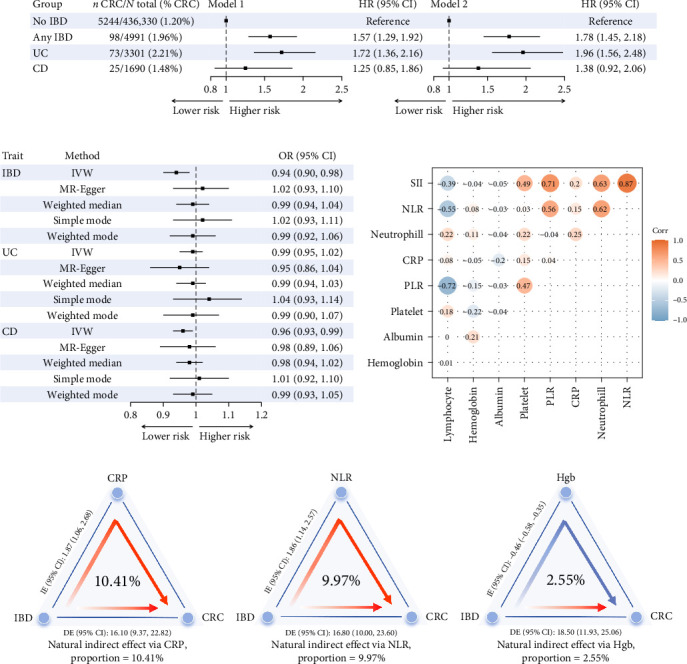
Prospective cohort, MR, and mediation analysis of IBD and CRC. (a) Associations between diagnosis for any IBD, UC, and CD and risk of CRC in UKB. (b) MR for the effect of genetic liability to IBD, CD, and UC on CRC. (c) Correlation heatmap of disease severity associated biomarkers. The color of the bubble corresponds to the beta coefficient of the association between disease severity associated biomarkers. Blue corresponds to a negative and red corresponds to a positive beta coefficient. The size of each bubble corresponds to the negative logarithm of the association *p* value, larger size corresponds to lower *p* values. (d) Mediation analysis of disease severity associated laboratory biomarkers on the association between IBD and CRC. CD, Crohn's disease; CI, confidence interval; CRC, colorectal cancer; CRP, C-reactive protein; DE, direct effect; Hgb, hemoglobin; HR, hazard ratio; IBD, inflammatory bowel disease; IE, indirect effect; MR, Mendelian randomization; NLR, neutrophil-to-lymphocyte ratio; PLR, platelet-to-lymphocyte ratio; SII, systemic immune-inflammation index; UC, ulcerative colitis; UKB, UK Biobank.

After adjusting for potential confounding factors (Model 2), IBD was associated with a 1.78-fold increased risk of CRC (95% CI: 1.45–2.18, *p* < 0.001). The adjusted HRs for CRC was 1.96 (95% CI: 1.56–2.48, *p* < 0.001) for UC and was 1.38 (95% CI: 0.92–2.06, *p*=0.115) for CD (Figure 2a). Subgroup analysis according to sex is shown in Table S5. Sensitivity analyses yielded results consistent with the main analysis (Table S6).

### 3.2. Study 2

We found no evidence of genetic correlations between IBD (*R*_g_: −0.11, 95% CI: −0.29 to 0.06, *p*=0.210), UC (*R*_g_: −0.08, 95% CI: −0.29 to 0.12, *p*=0.432), or CD (*R*_g_: −0.12, 95% CI: −0.31 to 0.08, *p*=0.236) and CRC (Table S7).

The mean *F* statistics of the IBD, UC, and CD instruments were 11.5, 11.4, and 16.5, respectively (Table S8). Evidence for the causal effect of IBD, UC, and CD on CRC risk was weak, and the direction of the effect estimates was inconsistent across sensitivity analyses (Figure 2b and Figure S2).

### 3.3. Study 3

CRP demonstrated a significant mediating effect, accounting for 10.41% of the total effect. The NLR also explained 9.97% of the total effect. Hgb showed a slight negative mediating effect, accounting for 2.55% of the total effect. Other biomarkers did not demonstrate mediating effects (Figure 2d and Table S9).

### 3.4. Study 4

After removing unknown genus, 197 GM taxa were finally included. Table S10 shows the potential causal effect of IBD on GM at different levels. For IBD, the disease status was significantly associated with decreased relative abundances of genus *Ruminococcus 2* (*β* = −9.463, *p*=0.0002) after Bonferroni's correction. Other suggestive positive associations were observed with the genera *Barnesiella*, *Olsenella*, and *Rikenellaceae RC9 gut group*. Conversely, negative associations were found with *Eubacterium ruminantium group*, *Phascolarctobacterium*, and *Ruminococcaceae UCG-003*. Horizontal pleiotropy and heterogeneity test results are shown in Table S11.

Our analysis also revealed significant associations between GM abundances and inflammatory biomarkers (Table 3 and Table S12). Two genera and one family were identified to be associated with both IBD and inflammatory biomarkers. The genus *Rikenellaceae RC9 gut group*, which was positively associated with IBD, showed a negative association with CRP levels (*β* = −0.019, *p*=0.011). The family Lactobacillaceae, negatively associated with CD, demonstrated a positive association with lymphocyte cell count (*β* = 0.023, *p*=0.042). The genus *Ruminococcus2*, which showed negative associations with both IBD and UC, was positively associated with neutrophil count (*β* = 0.028, *p*=0.038). Horizontal pleiotropy and heterogeneity test results are shown in Table S13.

### 3.5. Study 5

A total of 4991 IBD patients were included, of which 98 were subsequently diagnosed with CRC. Their baseline characteristics are detailed in Table S14.

The correlation analysis revealed strong positive relationships between SII and NLR (*r* = 0.87), NLR and neutrophil count (*r* = 0.62), and SII and neutrophil count (*r* = 0.63). Negative correlations were observed between PLR and lymphocyte count (*r* = −0.72) and between NLR and lymphocyte count (*r* = −0.55; Figure 2c). Highly correlated biomarkers and biomarkers with VIF exceeding five were removed (Table S4). Finally, the disease severity associated biomarker score were constructed using CRP, platelets, NLR, PLR, Hgb, and albumin.

**Figure 3:**
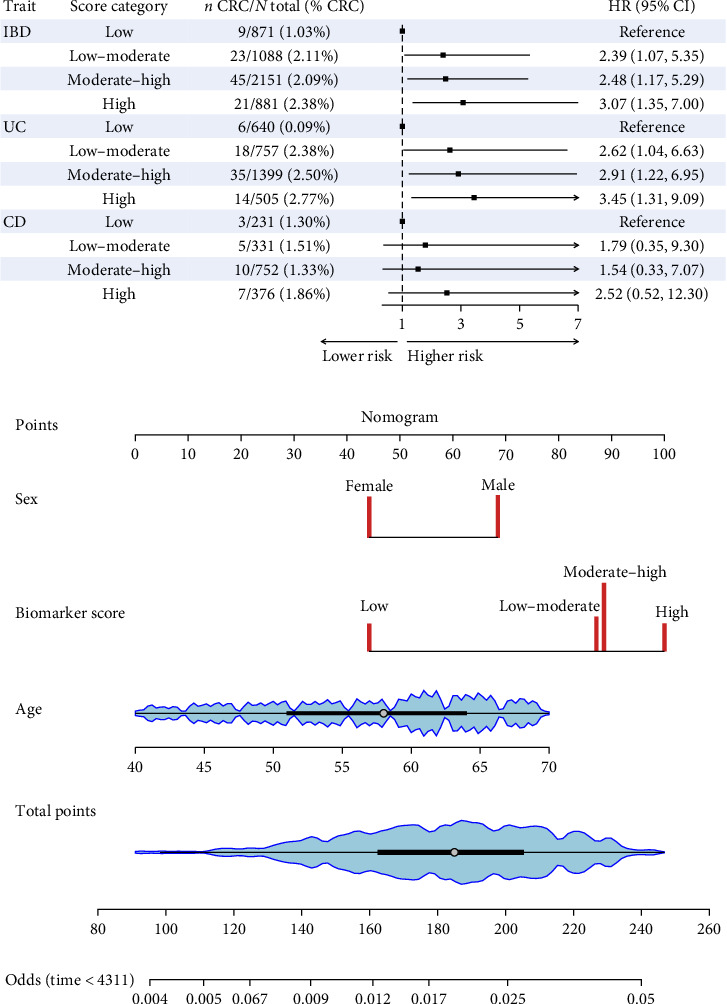
Association between IBD severity score and CRC risk. (a) Associations between disease severity associated laboratory biomarker score and risk of CRC in IBD, UC, and CD patients. (b) A nomogram integrated with three risk factors to predict the possibility of CRC in IBD patients. The scale of the line corresponding to each variable indicates the possible range of values, and the blue shading represents the density plot for continuous variables. The length of the line indicates the degree of the contribution of the factor to CRC risk in IBD patients. The Points at the top of the nomogram indicate the scores corresponding to different values of the risk factor, and the scores of all the variables are summed up to the total points. CD, Crohn's disease; CI, confidence interval; CRC, colorectal cancer; HR, hazard ratio; IBD, inflammatory bowel disease; UC, ulcerative colitis.

Compared to patients with low biomarker scores, IBD patients with low–moderate, moderate–high, and high scores had a 2.39- (95% CI: 1.07–5.35, *p*=0.034), 2.48- (95% CI: 1.17–5.29, *P*=0.018), and 3.07-times (95% CI: 1.35–7.00, *p*=0.008) higher risk of CRC, respectively (Figure 3a and Table S15).

Similarly, UC patients with low–moderate, moderate–high, and high biomarker scores had a 2.62- (95% CI: 1.04–6.63, *p*=0.041), 2.91- (95% CI: 1.22–6.95, *p*=0.016), and 3.45-times (95% CI: 1.31–9.09, *p*=0.012) higher CRC risk compared to patients with low biomarker scores. No statistically significant associations were observed among CD patients (Figure 3a and Table S15).

Age, sex, and the biomarker score were integrated into the nomogram (Figure 3b). Subgroup results are detailed in Table S16. Sensitivity analyses excluding indeterminate colitis yielded results consistent with the main analysis (Table S17).

## 4. Discussion

Our study provides novel insights into the complex interplays between IBD, GM, inflammatory biomarkers, and CRC risk. We demonstrated a significantly increased risk of CRC in IBD patients, and the underlying mechanism for this increased risk is not primarily driven by genetic predisposition. Instead, we found that inflammatory biomarkers play a crucial mediating role between IBD-induced GM dysbiosis and the development of CRC.

Our study revealed that individuals with IBD had a significantly higher risk of developing CRC, which align with previous findings [21, 22]. In our observational analysis, potential confounding factors have been fully adjusted; moreover, similar associations were observed in sensitivity analyses. This indicated the result was not likely to be biased by confounding or reverse causality. However, the genetic correlation analysis did not provide evidence of shared genetic architecture between IBD and CRC. Similarly, MR analysis did not reveal a causal relationship among the two traits. There are several possible reasons may explain this discrepancy (strong observational association, but no genetic correlation). First, we acknowledge that MR is subject to the biased effects from IVs selection. For instance, the genetic instruments (SNPs) used in our MR analysis, while strongly associated with IBD (*p* < 5 × 10^−8^), only account for a modest proportion of the total variation of the disease. The limited explanatory power of IVs may lead to reduced statistical power to detect causal effects. Second, while sensitivity analyses were conducted to assess the robustness of the causal estimates, the influence of horizontal pleiotropy cannot be entirely excluded. Actually, the discrepancy provides important insights into the IBD–CRC relationship: the increased CRC risk in IBD patients is likely driven by environmental and disease-related factors rather than shared genetic predisposition.

To further explore the underlying etiology between IBD and CRC, we performed a mediation analysis. We found CRP and NLR play significant mediating roles in the progression from IBD to CRC. As a key marker of chronic inflammation, CRP is primarily produced by hepatocytes in response to pro-inflammatory cytokines such as tumor necrosis factor-alpha (TNF-*α*), interleukin-6 (IL-6), and IL-1*β*. Studies have shown that CRP promotes colorectal carcinogenesis through multiple pathways. It contributes to an inflammatory microenvironment which can induce oxidative DNA damage and instability through the generation of reactive oxygen and nitrogen species, dysregulation of tumor suppressing genes like p53, and upregulate antiapoptotic protein genes [23]. The NLR is emerging as a predictive indicator that provides valuable insights into systemic inflammatory status and the balance between neutrophils and lymphocytes [24]. An elevated NLR indicates a disrupted equilibrium, characterized by relatively increased neutrophil count and decreased lymphocyte count, which can actively promote tumor progression. Neutrophils contribute to carcinogenesis through multiple mechanisms. They produce and release genotoxic substances that cause DNA damage, while simultaneously enhancing angiogenesis and creating an immunosuppressive environment [25]. Conversely, a reduced lymphocyte count may reflect impaired immune surveillance, allowing escape from immune-mediated tumor cell death [26].

Our MR analyses exploring the crosstalk among IBD, GM, and inflammatory biomarkers provided valuable insights into microbial-associated mechanisms of carcinogenesis linked to inflammatory pathways. The observed associations between IBD and alterations in specific gut microbial taxa, such as decreased abundance of genus *Ruminococcus 2* and family Lactobacillaceae, align with previous studies on GM dysbiosis in IBD [27–31]. Furthermore, the identified links between the abovementioned microbial taxa and inflammatory biomarkers suggest that GM dysbiosis contribute to the inflammatory status in IBD patients. Regarding genus *Ruminococcus* 2, it has a predominant role in producing cell-wall molecules and short-chain fatty acids (SCFAs) that modulate immune cell dynamics and granulopoiesis [32]. SCFAs have several beneficial actions, such as maintaining intestinal barrier function, regulating inflammatory and immune responses, and cell cycle processes [33]. Wang et al. [34] found that *Ruminococcus* 2 depletion was one of the alterations in gut microbiology in autoimmune diseases. As for clinical practice, a randomized trial of autologous fecal microbiota transplantation (auto-FMT) including *Ruminococcus* 2 has observed the higher counts of neutrophils, monocytes, and lymphocytes in auto-FMT-treated individuals [32]. Therapies correcting dysbiosis, including FMT and probiotics, maybe promising in IBD treatment. Previous studies have shown that the abundance of the genus *Rikenellaceae RC9 gut group* is reduced in IBD patients, which is in contrast to our findings [35]. It has been previously demonstrated that *Rikenellaceae* is a butyrate-producing bacteria capable of protecting the host from gut inflammation and IBD exacerbation [36]. This discrepancy highlights the complexity of microbiome dynamics in IBD and the abundance of *Rikenellaceae RC9 gut group* might vary at different stages of IBD.

We also discovered that patients with higher disease severity-associated biomarker score exhibited a significantly elevated risk of developing CRC. To date, the risk prediction model for IBD patients developing CRC is still lacking. The European Crohn's and Colitis Organization Guidelines Committee elaborate to stratify the risk of CRC in IBD patients based on clinical features (age and sex), comorbidities, and family history [37]. However, it does not include biomarkers such as CRP. Our findings underscore the practicality of using readily available and cost-effective biomarkers for individualized CRC risk stratification in IBD patients. We also developed a nomogram for the prediction of CRC in IBD patients, which incorporated seven parameters including age, sex, and the biomarker score constructed by CRP, NLR, PLR, Hgb, and albumin. All parameters are readily available in routine clinical examinations. Therefore, this nomogram will be useful for the risk assessment of CRC in IBD patients without the assistance of physicians. It is worth mentioning that the association was observed exclusively in patients with UC. The discrepancy in the risk of developing CRC between UC and CD may be attributed to variations in their inflammatory patterns, mucosal barrier dysfunction, genetic and epigenetic variations, immune responses, et cetera [38]. While UC characteristically presents with continuous colonic inflammation that creates a more consistent mutagenic environment, CD demonstrates a discontinuous, transmural inflammation across the gastrointestinal tract, potentially reducing uniform carcinogenic stimuli. It has also been found that the increase in the expression frequency of p53, the hypermethylation level of CpG islands of some genes such as mMLH1, and the activation of many cytokine pathways such as IL-6 and IL-11 are closely related to the occurrence of CRC in UC patients, which have not been observed in CD patients [39]. Moreover, the most commonly affected site of CD is small intestine, while colon is not prominent. Therefore, the overall risk of CRC in CD patients is lower than that in UC patients.

It is important to acknowledge several limitations in our study. First, the lack of factors such as endoscopic disease activity, medical therapy, and surveillance colonoscopy in the UKB constrains the evaluation of their effects on CRC risk. This may lead to potential confounding bias and affect inferences about the relationship between IBD and CRC. Second, the GWAS summary statistics of GM in MiBioGen is limited to the genus level, lacking more accurate species-level data. This may mask species-specific effects that could be critical in understanding CRC pathogenesis. Future research should prioritize high-resolution metagenomic approaches to capture more detailed microbial signatures. Third, the results were mainly derived from European population, which limits the generalizability of the results across diverse genetic and environmental backgrounds. Validations encompassing diverse ancestries are needed to establish the broader reproducibility of our findings.

Despite these limitations, to our knowledge, this is the first study to decipher the interplay among IBD, GM, and inflammatory biomarkers in the risk of CRC. These results contribute to our understanding of the potential mechanisms underlying the increased risk of CRC in IBD patients. However, it is important to note that further research is warranted to explore the in-depth mechanisms between IBD-associated GM dysbiosis and GM-associated inflammation and CRC.

## Figures and Tables

**Table 1:** Summary of the research question and data sources used, as well as key assumptions, strengths, and limitations of each method applied in the present study.

Method	Research question	Data sources	Key assumptions	Key strengths	Key limitations
Cox regression analysis	Are diagnoses of IBD associated with increased CRC risk?	UKB	Proportional hazards assumption; random censoring; linear relationship between log hazard and covariates	Large-scale population, prospective recording of data, low rate of loss to follow up, large availability of confounder data	Unmeasured confounding; reverse causation; measurement error

LDSC	Is there a shared genetic background between IBD and CRC?	GWAS summary data	Polygenicity; no population stratification	Using GWAS summary data maximizes sample sizes and power; indicating genetic correlation between traits	Cannot assess causality

MR	Does genetic liability to IBD have a causal effect on CRC?	GWAS summary data and individual genotype data in UKB	Genetic variants are: (1) robustly associated with exposure; (2) exclusively associated with outcome through exposure; (3) independent of any confounders	Using genetic variants as instrument variables allows the assessment of causal effects; avoiding unmeasured confounding bias and reverse causation	Pleiotropy; weak instrument bias; assortative mating; canalization; dynastic effects

Mediation analysis	Do disease severity associated laboratory biomarkers mediate between IBD and CRC?	UKB	No unmeasured confounders for: (1) exposure–outcome relationship; (2) mediator–outcome relationship; (3) exposure–mediator relationship	Testing the mechanisms/pathways by which exposure affects outcome through mediators; quantifying direct and indirect effects separately	Cannot establish causality or make strong causal inferences; highly sensitive to model specification

Abbreviations: CRC, colorectal cancer; GWAS, genome wide association study; IBD, inflammatory bowel disease; LDSC, linkage disequilibrium score regression; MR, Mendelian randomization; UKB, UK Biobank.

**Table 2:** Baseline characteristics of the study participants in the UKB.

Characteristic^a^	Non-IBD (*N* = 436,330)	IBD (*N* = 4,991)	*p*-Value^b^
Age at baseline (years), mean (SD)	56.2 (8.1)	56.9 (8.0)	<0.001
Sex, *n* (%)	—	—	0.007
Female	231,993 (53.2%)	2558 (51.3%)	—
Male	204,337 (46.8%)	2433 (48.7%)	—
Ethnicity, *n* (%)	—	—	<0.001
White	409,333 (93.8%)	4758 (95.3%)	—
Asian	10,386 (2.4%)	118 (2.4%)	—
African	7212 (1.7%)	37 (0.7%)	—
Mixed background	2597 (0.6%)	17 (0.3%)	—
Unknown	6802 (1.6%)	61 (1.2%)	—
Education background, *n* (%)	—	—	<0.001
College/university degree	358,108 (82.1%)	3927 (78.7%)	—
Non-college/university degree	72,605 (16.6%)	1011 (20.3%)	—
Unknown	5617 (1.3%)	53 (1.1%)	—
Townsend deprivation index	−1.29 (3.10)	−1.25 (3.09)	0.215
BMI (kg/m^2^), *n* (%)	—	—	<0.001
18.5–24.9	108,704 (24.9%)	1124 (22.5%)	—
24.9–29.9	187,192 (42.9%)	2165 (43.4%)	—
>29.9	136,499 (31.3%)	1643 (32.9%)	—
Unknown	3935 (0.9%)	59 (1.2%)	—
Smoking status, *n* (%)	—	—	<0.001
Never	46,008 (10.5%)	479 (9.6%)	—
Past	148,260 (34.0%)	2127 (42.6%)	—
Current	239,842 (55.0%)	2365 (47.4%)	—
Unknown	2220 (0.5%)	20 (0.4%)	—
Family CRC history of parents and siblings, *n* (%)	—	—	0.411
Yes	41,020 (9.4%)	473 (9.5%)	—
No	353,804 (81.1%)	4071 (81.6%)	—
Unknown	41,506 (9.5%)	447 (9.0%)	—
CRC screening history, *n* (%)	—	—	<0.001
Yes	276,779 (63.4%)	636 (12.7%)	—
No	7054 (1.6%)	76 (1.5%)	—
Unknown	152,497 (34.9%)	4279 (85.7%)	—
WCRF/AICR HLI score, mean (SD)	3.08 (0.91)	2.98 (0.90)	<0.001
Incident CRC, *n* (%)	5244 (1.2%)	98 (2.0%)	<0.001

Abbreviations: BMI, body mass index; CRC, colorectal cancer; HLI, healthy lifestyle index; IBD, inflammatory bowel disease; UKB, UK Biobank; WCRF/AICR, the World Cancer Research Fund/American Institute for Cancer Research.

^a^Mean (SD) values and *n* (%) are reported for continuous and categorical variables, respectively.

^b^ *t* test and Pearson's *χ*^2^ test are tested for continuous and categorical variables, respectively.

**Table 3:** Potential causal effects of IBD on GM and GM on inflammatory biomarkers.

Exposure	Outcome	SNPs	*β*	SE	*p*
Association between IBD and GM					
IBD	genus_*Rikenellaceae RC9 gut group*	12	11.595	5.833	0.047
CD	family_*Lactobacillaceae*	72	−0.031	0.015	0.037
IBD	genus_*Ruminococcus 2*	13	−9.463	2.560	<0.001
UC	genus_*Ruminococcus 2*	9	−11.999	4.094	0.003
Association between GM and biomarkers					
genus_*Rikenellaceae RC9 gut group*	CRP	6	−0.019	0.007	0.011
family_*Lactobacillaceae*	Lymphocyte count	4	0.023	0.011	0.042
genus_*Ruminococcus 2*	Neutrophil count	7	0.028	0.013	0.038

Abbreviations: CD, Crohn's disease; CRP, C-reactive protein; GM, gut microbiota; IBD, inflammatory bowel disease; SE, standard error; SNP, single nucleotide polymorphism; UC, ulcerative colitis.

## Data Availability

The data used in this study are available via UK Biobank on request (http://ukbiobank.ac.uk/register-apply/). GWAS summary statistics for IBD, UC, CD, and CRC used in the LDSC and MR analyses are publicly available in GWAS catalog (https://www.ebi.ac.uk/gwas/home/).

## Supporting Information

Supporting Information Table S1: Healthy lifestyle index components in UK Biobank. Table S2: Harmonized dataset for the two-sample MR analyses investigating causal effects of genetic liability to IBD, UC, and CD on CRC. Table S3: Sources of GWAS summary statistics. Table S4: Variance inflation factor (VIF) results for multivariate model incorporating different disease severity associated laboratory biomarkers. Table S5: Associations between diagnosis for any IBD, UC, and CD and risk of CRC by sex. Table S6: Sensitivity analyses of associations between diagnosis for any IBD, UC, and CD and risk of CRC. Table S7: Genetic correlation between the liability to IBD, UC, and CD and CRC. Table S8: *F* statistic and power calculation for MR analyses for IBD, UC, and CD in relation to CRC. Table S9: Mediation analysis of the disease severity associated laboratory biomarkers for the association between IBD and CRC. Table S10: Association between IBD and GM using inverse variance weighted method. Table S11: Heterogeneity and pleiotropy test between IBD and GM. Table S12: Association between GM and biomarkers using inverse variance weighted method. Table S13: Heterogeneity and pleiotropy test between GM and biomarkers. Table S14: Baseline characteristics of IBD participants by incident CRC in UK Biobank. Table S15: Associations between disease severity associated laboratory biomarker score and risk of CRC in IBD, UC, and CD patients. Table S16: Associations between disease severity associated laboratory biomarker score and risk of CRC in IBD, UC, and CD patients by sex. Table S17: Sensitivity analysis of the associations between disease severity associated laboratory biomarker score and risk of CRC in IBD, UC, and CD patients excluding dual diagnosis. Figure S1: Flowchart of the participants excluded from the cohort study. Figure S2: Association between UC and CD and CRC outcomes based on two step MR analysis.
