# Supplementary material for: Deciphering the Interplay Among Inflammatory Bowel Disease, Gut Microbiota, and Inflammatory Biomarkers in the Risk of Colorectal Cancer
Source: Mediators Inflamm. 2025 Mar 8;2025:4967641. doi: 10.1155/mi/4967641 (PMC11986182; doi:10.1155/mi/4967641)
Supplement: Supporting Information — Table S1: Healthy lifestyle index components in UK Biobank. Table S2: Harmonized dataset for the two-sample MR analyses investigating causal effects of genetic liability to IBD, UC, and CD on CRC. Table S3: Sources of GWAS summary statistics. Table S4: Variance inflation factor (VIF) results for multivariate model incorporating different disease severity associated laboratory biomarkers. Table S5: Associations between diagnosis for any IBD, UC, and CD and risk of CRC by sex. Table S6: Sensitivity analyses of associations between diagnosis for any IBD, UC, and CD and risk of CRC. Table S7: Genetic correlation between the liability to IBD, UC, and CD and CRC. Table S8: F statistic and power calculation for MR analyses for IBD, UC, and CD in relation to CRC. Table S9: Mediation analysis of the disease severity associated laboratory biomarkers for the association between IBD and CRC. Table S10: Association between IBD and GM using inverse variance weighted method. Table S11: Heterogeneity and pleiotropy test between IBD and GM. Table S12: Association between GM and biomarkers using inverse variance weighted method. Table S13: Heterogeneity and pleiotropy test between GM and biomarkers. Table S14: Baseline characteristics of IBD participants by incident CRC in UK Biobank. Table S15: Associations between disease severity associated laboratory biomarker score and risk of CRC in IBD, UC, and CD patients. Table S16: Associations between disease severity associated laboratory biomarker score and risk of CRC in IBD, UC, and CD patients by sex. Table S17: Sensitivity analysis of the associations between disease severity associated laboratory biomarker score and risk of CRC in IBD, UC, and CD patients excluding dual diagnosis. Figure S1: Flowchart of the participants excluded from the cohort study. Figure S2: Association between UC and CD and CRC outcomes based on two step MR analysis. [file 4967641.f1.docx]

# **Deciphering the interplay among inflammatory bowel disease, gut microbiota and inflammatory biomarkers in the risk of colorectal cancer**

**Supplementary Tables Legends**

Supplementary Table 1. Healthy lifestyle index components in UK Biobank.

Supplementary Table 2. Harmonised dataset for the two-sample MR analyses investigating causal effects of genetic liability to IBD, UC, CD on CRC.

Supplementary Table 3. Sources of GWAS summary statistics.

Supplementary Table 4. Variance inflation factor (VIF) results for multivariate model incorporating different disease severity associated laboratory biomarkers.

Supplementary Table 5. Associations between diagnosis for any IBD, UC, CD and risk of CRC by sex.

Supplementary Table 6. Sensitivity analyses of associations between diagnosis for any IBD, UC, CD and risk of CRC.

Supplementary Table 7. Genetic correlation between the liability to IBD, UC, and CD and CRC.

Supplementary Table 8. F statistic and power calculation for Mendelian randomization analyses for IBD, UC, CD in relation to CRC.

Supplementary Table 9. Mediation analysis of the disease severity associated laboratory biomarkers for the association between IBD and CRC.

Supplementary Table 10. Association between IBD and GM using inverse variance weighted method.

Supplementary Table 11. Heterogeneity and pleiotropy test between IBD and GM.

Supplementary Table 12. Association between GM and biomarkers using inverse variance weighted method.

Supplementary Table 13. Heterogeneity and pleiotropy test between GM and biomarkers.

Supplementary Table 14. Baseline characteristics of IBD participants by incident CRC in UK Biobank.

Supplementary Table 15. Associations between disease severity associated laboratory biomarker score and risk of CRC in IBD, UC, CD patients.

Supplementary Table 16. Associations between disease severity associated laboratory biomarker score and risk of CRC in IBD, UC, CD patients by sex.

Supplementary Table 17. Sensitivity analysis of the associations between disease severity associated laboratory biomarker score and risk of CRC in IBD, UC, CD patients excluding dual diagnosis.

## Supplementary Table 1. Healthy lifestyle index components in UK Biobank.

| **WCRF/AICR recommendations** | **HLI components** | **Categories** | **Score** | **UK Biobank Field ID** |
| --- | --- | --- | --- | --- |
| 1) Eat a diet rich in wholegrains, vegetables, fruit and beans^1^ | Total fruit and vegetable intake^2^ | <3 serving/day  ≥3–<5 servings/day  ≥5 servings/day | 0  0.25  0.5 | 1289, 1299, 1309, 1319 |
|  | Whole grains intake^3^ | ≤2 serving/day  >2–<5.5 servings/day  ≥5.5 servings/day | 0  0.25  0.5 | 1438, 1448, 1458, 1468 |
| 2) Limit consumption of red and processed meat | Red meat intake | >4 times/week  2–4 times/week  ≤once/week | 0  0.25  0.5 | 1369, 1379, 1389 |
|  | Processed meat intake | >4 times/week  2–4 times/week  ≤once/week | 0  0.25  0.5 | 1349 |
| 3) Limit alcohol consumption | Alcohol intake (g/day)^4^ | 0.0  >0.0–≤14.0 (female),>0.0–≤28.0 (male)  >14.0 (female), >28.0 (male) | 1  0.5  0 | 1588, 1578, 1608, 1568, 1598, 5364 |
| 4) Be physically active | Total moderate-vigorous physical activity (min/wk)^5^ | <75  75–<150  ≥150 | 0  0.5  1 | 884, 894, 904, 914 |
| 5) Be a healthy weight | BMI (kg/m^2^) | 18.5–24.9  25.0–29.9  >29.9 | 0.5  0.25  0 | 21001 |
|  | Waist Circumference (cm) | <80 (female), <94 (male)  >80–<88 (female), >94–<102  ≥88 (female), ≥102 (male) | 0.5  0.25  0 | 48 |
| 6) Smoking is not included in the WCRF/AICR guidelines | Smoking | Never smoked  Former smoker  Current smoker | 1  0.5  0 | 20116, 20161 |

^1^ The UK Biobank food frequency questionnaire does not include information on intake of beans.

^2^ Amount per serving: fresh fruit -1 piece, dried fruit -5 pieces, cooked/raw vegetables -3 heaped tablespoons.

^3^ Amount per serving: Bran/oat/muesli cereal -1 bowl/day, Whole-meal/wholegrain bread -1 slice/day.

^4^ Glasses were converted to UK units as follows: red or white wine = 1.7 units, fortified wine=1.2 units, pint = 2.4 units, spirits = 1 unit, other (e.g. alcopops) =1.2 units. Amounts were also converted to grams pure ethanol (1 UK unit=8g) to aid international comprehension and comparison.

^5^ Physical activity (moderate and vigorous) was quantified by multiplying the number of days per week of each physical activity by duration (minutes/day) and summing the moderate and vigorous physical activity.

Abbreviations: WCRF/AICR: the World Cancer Research Fund/American Institute for Cancer Research, HLI: healthy lifestyle index, BMI, body mass index.

## Supplementary Table 2. Harmonised dataset for the two-sample MR analyses investigating causal effects of genetic liability to IBD, UC, CD on CRC.

| SNP | Effect allele. IBD | Other allele. IBD | Effect allele. CRC | Other allele. CRC | Beta. IBD | Beta. CRC | SE. IBD | pval. IBD | SE. CRC | pval. CRC | Removed? |
| --- | --- | --- | --- | --- | --- | --- | --- | --- | --- | --- | --- |
| IBD |  |  |  |  |  |  |  |  |  |  |  |
| rs10055349 | A | G | A | G | 0.1038 | -0.0149 | 0.0148 | 2.1650E-12 | 0.0209 | 4.7484E-01 | No |
| rs10114470 | C | T | C | T | 0.1475 | -0.0005 | 0.0137 | 4.0980E-27 | 0.0187 | 9.7884E-01 | No |
| rs10761659 | G | A | G | A | 0.1585 | 0.0023 | 0.0126 | 2.2980E-36 | 0.0170 | 8.9272E-01 | No |
| rs10800309 | G | A | G | A | -0.1230 | 0.0044 | 0.0133 | 1.9420E-20 | 0.0182 | 8.0905E-01 | No |
| rs10826797 | T | G | T | G | -0.0990 | 0.0322 | 0.0136 | 3.9860E-13 | 0.0194 | 9.7446E-02 | No |
| rs10953551 | G | A | G | A | -0.1033 | 0.0066 | 0.0127 | 4.9430E-16 | 0.0174 | 7.0524E-01 | No |
| rs11066188 | A | G | A | G | 0.0874 | -0.0793 | 0.0130 | 1.7590E-11 | 0.0172 | 3.9747E-06 | No |
| rs111456533 | A | G | A | G | -0.1031 | -0.0164 | 0.0170 | 1.1790E-09 | 0.0228 | 4.7043E-01 | No |
| rs11152949 | G | A | G | A | 0.1019 | 0.0067 | 0.0133 | 1.5630E-14 | 0.0187 | 7.1859E-01 | No |
| rs11195128 | T | C | T | C | 0.0792 | -0.0434 | 0.0133 | 2.7440E-09 | 0.0182 | 1.6956E-02 | No |
| rs11221335 | C | T | C | T | 0.0827 | -0.0320 | 0.0148 | 2.4400E-08 | 0.0202 | 1.1379E-01 | No |
| rs11236797 | A | C | A | C | 0.1488 | 0.0092 | 0.0125 | 7.1880E-33 | 0.0170 | 5.8676E-01 | No |
| rs112874012 | T | C | T | C | -0.1991 | -0.0239 | 0.0318 | 3.9020E-10 | 0.0425 | 5.7484E-01 | No |
| rs112936798 | C | A | C | A | -0.1844 | -0.1300 | 0.0332 | 2.8890E-08 | 0.0595 | 2.8960E-02 | No |
| rs1131095 | C | T | C | T | 0.1635 | -0.0040 | 0.0131 | 1.2190E-35 | 0.0182 | 8.2648E-01 | No |
| rs1143687 | T | C | T | C | -0.1382 | -0.0399 | 0.0251 | 3.8290E-08 | 0.0328 | 2.2479E-01 | No |
| rs11548656 | G | A | G | A | -0.2374 | -0.0171 | 0.0362 | 5.1840E-11 | 0.0468 | 7.1510E-01 | No |
| rs1157509 | G | A | G | A | 0.1449 | 0.0307 | 0.0172 | 3.3490E-17 | 0.0228 | 1.7684E-01 | No |
| rs11576006 | C | T | C | T | -0.0872 | 0.0122 | 0.0154 | 1.4880E-08 | 0.0205 | 5.5171E-01 | No |
| rs11581607 | A | G | A | G | -0.6578 | 0.0066 | 0.0294 | 4.5900E-111 | 0.0345 | 8.4821E-01 | No |
| rs11669299 | T | C | T | C | -0.1107 | -0.0374 | 0.0157 | 1.8360E-12 | 0.0212 | 7.7552E-02 | No |
| rs116760029 | A | G | A | G | 0.1842 | -0.0220 | 0.0311 | 3.1290E-09 | 0.0471 | 6.4109E-01 | No |
| rs11677002 | C | T | C | T | -0.0931 | 0.0109 | 0.0126 | 1.3710E-13 | 0.0175 | 5.3306E-01 | No |
| rs117292830 | A | G | A | G | 0.3398 | -0.0565 | 0.0394 | 6.6860E-18 | 0.0470 | 2.2935E-01 | No |
| rs11734570 | A | G | A | G | 0.0694 | -0.0174 | 0.0127 | 4.8010E-08 | 0.0172 | 3.1290E-01 | No |
| rs11768365 | G | A | G | A | -0.0837 | -0.0134 | 0.0152 | 3.8790E-08 | 0.0213 | 5.2761E-01 | No |
| rs117746331 | T | C | T | C | -0.1886 | 0.0286 | 0.0346 | 4.9900E-08 | 0.0443 | 5.1838E-01 | No |
| rs117981694 | A | G | A | G | 0.3452 | -0.0198 | 0.0411 | 4.5320E-17 | 0.0495 | 6.8961E-01 | No |
| rs11870407 | A | C | A | C | -0.1010 | -0.0090 | 0.0140 | 4.8250E-13 | 0.0189 | 6.3515E-01 | No |
| rs12136659 | C | T | C | T | 0.0870 | -0.0831 | 0.0142 | 1.0200E-09 | 0.0196 | 2.1788E-05 | No |
| rs12206377 | G | A | G | A | 0.2504 | 0.0042 | 0.0416 | 1.7160E-09 | 0.0455 | 9.2628E-01 | No |
| rs12411216 | C | A | C | A | 0.0830 | 0.0000 | 0.0126 | 3.8530E-11 | 0.0171 | 9.9814E-01 | No |
| rs1250573 | A | G | A | G | -0.0980 | 0.0904 | 0.0138 | 1.1070E-12 | 0.0190 | 2.0574E-06 | No |
| rs1265098 | C | T | C | T | -0.1278 | 0.0150 | 0.0157 | 4.2540E-16 | 0.0208 | 4.6877E-01 | No |
| rs1268339 | C | T | C | T | 0.0907 | 0.0325 | 0.0163 | 2.7520E-08 | 0.0218 | 1.3604E-01 | No |
| rs12720356 | C | A | C | A | 0.1585 | -0.0024 | 0.0214 | 1.4420E-13 | 0.0298 | 9.3485E-01 | No |
| rs12825700 | A | G | A | G | 0.1324 | -0.0147 | 0.0127 | 1.2750E-25 | 0.0177 | 4.0617E-01 | No |
| rs12936409 | T | C | T | C | 0.1406 | -0.0038 | 0.0124 | 7.7260E-30 | 0.0170 | 8.2435E-01 | No |
| rs1297264 | G | A | G | A | -0.1462 | 0.0097 | 0.0126 | 3.9800E-31 | 0.0172 | 5.7181E-01 | No |
| rs1317209 | A | G | A | G | 0.1164 | -0.0110 | 0.0160 | 3.7860E-13 | 0.0214 | 6.0529E-01 | No |
| rs1336900 | A | G | A | G | -0.0848 | -0.0164 | 0.0128 | 2.9840E-11 | 0.0174 | 3.4603E-01 | No |
| rs13422838 | C | T | C | T | -0.1143 | -0.0184 | 0.0205 | 2.5570E-08 | 0.0287 | 5.2155E-01 | No |
| rs1445004 | T | C | T | C | 0.1689 | -0.0613 | 0.0127 | 3.4840E-40 | 0.0174 | 4.3566E-04 | No |
| rs145126485 | C | A | C | A | 0.2464 | 0.0144 | 0.0356 | 4.7730E-12 | 0.0494 | 7.7041E-01 | No |
| rs145568234 | G | T | G | T | 0.8600 | -0.0398 | 0.0476 | 4.7310E-73 | 0.0754 | 5.9808E-01 | No |
| rs1456896 | T | C | T | C | 0.0879 | -0.0107 | 0.0133 | 4.5040E-11 | 0.0184 | 5.6017E-01 | No |
| rs150249594 | T | C | T | C | 0.7528 | 0.1736 | 0.1248 | 1.6170E-09 | 0.1266 | 1.7024E-01 | No |
| rs154873 | A | G | A | G | -0.0813 | -0.0004 | 0.0132 | 7.3770E-10 | 0.0184 | 9.8397E-01 | No |
| rs1558619 | T | G | T | G | -0.0843 | 0.0224 | 0.0123 | 8.8970E-12 | 0.0169 | 1.8348E-01 | No |
| rs16940202 | C | T | C | T | 0.1130 | 0.0204 | 0.0169 | 2.5050E-11 | 0.0219 | 3.5033E-01 | No |
| rs17656349 | T | C | T | C | 0.0731 | 0.0021 | 0.0125 | 5.1670E-09 | 0.0172 | 9.0344E-01 | No |
| rs17800987 | G | A | G | A | 0.1843 | -0.0228 | 0.0222 | 1.0660E-16 | 0.0272 | 4.0349E-01 | No |
| rs1864239 | G | A | G | A | 1.3366 | -0.0407 | 0.1782 | 6.2710E-14 | 0.1272 | 7.4933E-01 | No |
| rs194746 | T | C | T | C | 0.0833 | -0.0010 | 0.0124 | 1.8360E-11 | 0.0171 | 9.5536E-01 | No |
| rs212402 | A | G | A | G | -0.0743 | -0.0243 | 0.0130 | 1.0590E-08 | 0.0180 | 1.7790E-01 | No |
| rs2229092 | C | A | C | A | 0.1463 | 0.0485 | 0.0267 | 4.3120E-08 | 0.0369 | 1.8828E-01 | No |
| rs2284553 | G | A | G | A | 0.0742 | -0.0149 | 0.0128 | 7.4010E-09 | 0.0179 | 4.0675E-01 | No |
| rs2301127 | A | G | A | G | 0.0783 | -0.0235 | 0.0126 | 4.9610E-10 | 0.0171 | 1.7026E-01 | No |
| rs2384352 | G | A | G | A | 0.0951 | 0.0229 | 0.0131 | 3.1180E-13 | 0.0181 | 2.0560E-01 | No |
| rs2413583 | T | C | T | C | -0.1732 | 0.0193 | 0.0171 | 4.5950E-24 | 0.0225 | 3.8984E-01 | No |
| rs243505 | G | A | G | A | -0.0805 | -0.0077 | 0.0128 | 3.0380E-10 | 0.0174 | 6.5832E-01 | No |
| rs2816972 | G | A | G | A | 0.1107 | 0.0295 | 0.0202 | 3.8960E-08 | 0.0269 | 2.7232E-01 | No |
| rs2836881 | T | G | T | G | -0.1643 | -0.0135 | 0.0146 | 1.9590E-29 | 0.0192 | 4.8170E-01 | No |
| rs28374519 | A | G | A | G | -0.1105 | -0.0049 | 0.0137 | 6.5470E-16 | 0.0186 | 7.9161E-01 | No |
| rs2838517 | C | T | C | T | -0.1280 | 0.0161 | 0.0125 | 1.8350E-24 | 0.0175 | 3.5697E-01 | No |
| rs2961704 | T | C | T | C | -0.1459 | 0.0189 | 0.0227 | 1.3110E-10 | 0.0304 | 5.3446E-01 | No |
| rs3024493 | A | C | A | C | 0.1911 | 0.0209 | 0.0165 | 4.0410E-31 | 0.0237 | 3.7642E-01 | No |
| rs3094571 | A | G | A | G | -0.0839 | 0.0211 | 0.0146 | 8.2230E-09 | 0.0172 | 2.1996E-01 | No |
| rs341295 | T | C | T | C | 0.0702 | 0.0066 | 0.0124 | 1.4510E-08 | 0.0169 | 6.9459E-01 | No |
| rs34236350 | T | C | T | C | 0.1317 | 0.0128 | 0.0164 | 1.0180E-15 | 0.0236 | 5.8753E-01 | No |
| rs35171809 | G | A | G | A | 0.1088 | 0.0256 | 0.0123 | 1.1560E-18 | 0.0170 | 1.3116E-01 | No |
| rs3792111 | T | C | T | C | 0.1391 | -0.0063 | 0.0124 | 5.1220E-29 | 0.0171 | 7.1134E-01 | No |
| rs3820328 | G | A | G | A | -0.0926 | -0.0088 | 0.0129 | 8.3110E-13 | 0.0177 | 6.1854E-01 | No |
| rs3829110 | G | A | G | A | 0.1574 | 0.0050 | 0.0125 | 3.5200E-36 | 0.0175 | 7.7445E-01 | No |
| rs3850378 | C | T | C | T | 0.1536 | -0.0013 | 0.0207 | 1.1000E-13 | 0.0304 | 9.6541E-01 | No |
| rs3897234 | C | T | C | T | 0.0971 | -0.0111 | 0.0145 | 1.8990E-11 | 0.0201 | 5.8209E-01 | No |
| rs395157 | T | C | T | C | 0.0776 | -0.0225 | 0.0124 | 4.6300E-10 | 0.0169 | 1.8442E-01 | No |
| rs4072601 | A | G | A | G | -0.1264 | 0.0215 | 0.0177 | 8.4330E-13 | 0.0234 | 3.5932E-01 | No |
| rs4236540 | T | G | T | G | -0.0810 | -0.0146 | 0.0139 | 5.5440E-09 | 0.0191 | 4.4619E-01 | No |
| rs4256018 | G | T | G | T | 0.0786 | -0.0195 | 0.0138 | 1.2300E-08 | 0.0188 | 3.0015E-01 | No |
| rs4380956 | A | G | A | G | 0.0907 | 0.0145 | 0.0127 | 1.1200E-12 | 0.0176 | 4.0836E-01 | No |
| rs4710973 | C | T | C | T | -0.0842 | -0.0056 | 0.0132 | 1.6730E-10 | 0.0177 | 7.5083E-01 | No |
| rs4807569 | C | A | C | A | 0.1281 | -0.0111 | 0.0152 | 4.2390E-17 | 0.0214 | 6.0322E-01 | No |
| rs4845604 | A | G | A | G | -0.1388 | -0.0091 | 0.0185 | 7.0940E-14 | 0.0242 | 7.0776E-01 | No |
| rs503734 | G | A | G | A | -0.0692 | -0.0122 | 0.0124 | 2.6740E-08 | 0.0172 | 4.7766E-01 | No |
| rs55946629 | A | C | A | C | 0.1298 | -0.0619 | 0.0180 | 5.4540E-13 | 0.0260 | 1.7230E-02 | No |
| rs56062135 | T | C | T | C | 0.1382 | 0.0197 | 0.0145 | 1.3710E-21 | 0.0203 | 3.3088E-01 | No |
| rs56116661 | T | C | T | C | -0.1000 | 0.0421 | 0.0163 | 9.2710E-10 | 0.0216 | 5.1247E-02 | No |
| rs5754100 | C | T | C | T | 0.1293 | 0.0172 | 0.0160 | 7.1400E-16 | 0.0219 | 4.3208E-01 | No |
| rs59043219 | A | G | A | G | 0.0738 | -0.0073 | 0.0129 | 1.0900E-08 | 0.0177 | 6.8115E-01 | No |
| rs6017342 | C | A | C | A | 0.1156 | -0.0365 | 0.0135 | 1.0710E-17 | 0.0184 | 4.6756E-02 | No |
| rs6062496 | A | G | A | G | 0.1370 | -0.0458 | 0.0129 | 2.8260E-26 | 0.0173 | 8.1964E-03 | No |
| rs6063502 | G | A | G | A | -0.0734 | 0.0294 | 0.0134 | 4.5480E-08 | 0.0176 | 9.4795E-02 | No |
| rs62183956 | T | C | T | C | -0.0780 | 0.0356 | 0.0125 | 4.4890E-10 | 0.0173 | 3.9200E-02 | No |
| rs62228374 | A | G | A | G | 0.2669 | 0.0861 | 0.0445 | 2.0040E-09 | 0.0592 | 1.4616E-01 | No |
| rs62324212 | A | C | A | C | 0.0886 | -0.0289 | 0.0127 | 2.6740E-12 | 0.0175 | 9.9646E-02 | No |
| rs62408218 | T | C | T | C | -0.0818 | 0.0182 | 0.0129 | 2.4020E-10 | 0.0180 | 3.1197E-01 | No |
| rs62482552 | A | G | A | G | -0.0737 | 0.0094 | 0.0131 | 1.9710E-08 | 0.0178 | 5.9605E-01 | No |
| rs6584282 | G | A | G | A | -0.1520 | 0.0420 | 0.0124 | 1.1940E-34 | 0.0170 | 1.3215E-02 | No |
| rs6674040 | T | G | T | G | -0.1129 | -0.0014 | 0.0124 | 6.3070E-20 | 0.0170 | 9.3665E-01 | No |
| rs67289879 | T | C | T | C | 0.0898 | -0.0216 | 0.0162 | 3.0410E-08 | 0.0211 | 3.0586E-01 | No |
| rs6740847 | G | A | G | A | -0.0924 | -0.0031 | 0.0125 | 1.2180E-13 | 0.0170 | 8.5494E-01 | No |
| rs687308 | T | C | T | C | -0.2294 | 0.0690 | 0.0174 | 1.1330E-39 | 0.0223 | 1.9860E-03 | No |
| rs6933404 | C | T | C | T | 0.0863 | 0.0108 | 0.0149 | 6.6410E-09 | 0.0215 | 6.1694E-01 | No |
| rs7190426 | C | A | C | A | -0.0872 | -0.0012 | 0.0155 | 2.0570E-08 | 0.0207 | 9.5396E-01 | No |
| rs72748445 | A | C | A | C | -0.1100 | 0.0974 | 0.0141 | 5.3720E-15 | 0.0188 | 2.1681E-07 | No |
| rs72852162 | C | A | C | A | -0.1129 | -0.0033 | 0.0202 | 2.2980E-08 | 0.0279 | 9.0547E-01 | No |
| rs744166 | G | A | G | A | -0.1109 | 0.0212 | 0.0126 | 1.3400E-18 | 0.0172 | 2.1767E-01 | No |
| rs749910 | A | G | A | G | 0.1961 | -0.0001 | 0.0138 | 7.8280E-46 | 0.0202 | 9.9469E-01 | No |
| rs7532133 | G | A | G | A | 0.0789 | -0.0105 | 0.0134 | 3.8320E-09 | 0.0182 | 5.6514E-01 | No |
| rs755374 | T | C | T | C | 0.1767 | 0.0168 | 0.0134 | 1.5860E-39 | 0.0195 | 3.8723E-01 | No |
| rs7608697 | C | A | C | A | 0.1395 | 0.0068 | 0.0126 | 1.6710E-28 | 0.0173 | 6.9484E-01 | No |
| rs76286777 | C | T | C | T | 0.0996 | 0.0017 | 0.0151 | 4.6550E-11 | 0.0214 | 9.3639E-01 | No |
| rs76527535 | T | C | T | C | -0.0864 | -0.0157 | 0.0156 | 2.8710E-08 | 0.0205 | 4.4337E-01 | No |
| rs7741847 | A | G | A | G | 0.1588 | 0.0018 | 0.0244 | 7.0830E-11 | 0.0319 | 9.5406E-01 | No |
| rs78703675 | A | G | A | G | 0.1078 | 0.0053 | 0.0187 | 7.6200E-09 | 0.0232 | 8.1899E-01 | No |
| rs78771661 | T | C | T | C | -0.3848 | 0.0545 | 0.0669 | 8.9490E-09 | 0.0867 | 5.2971E-01 | No |
| rs7918084 | T | C | T | C | 0.0710 | -0.0307 | 0.0125 | 1.3830E-08 | 0.0173 | 7.6352E-02 | No |
| rs7995004 | T | C | T | C | 0.0833 | -0.0368 | 0.0148 | 1.7870E-08 | 0.0198 | 6.2764E-02 | No |
| rs80262450 | A | G | A | G | 0.1581 | 0.0212 | 0.0190 | 1.0410E-16 | 0.0277 | 4.4466E-01 | No |
| rs938650 | A | G | A | G | -0.1074 | -0.0312 | 0.0189 | 1.4130E-08 | 0.0252 | 2.1599E-01 | No |
| rs9934775 | T | C | T | C | -0.1116 | 0.0469 | 0.0172 | 8.7670E-11 | 0.0230 | 4.1158E-02 | No |
| rs10052709 | G | C | G | C | -0.1236 | 0.0384 | 0.0187 | 3.4420E-11 | 0.0240 | 1.1013E-01 | Yes |
| rs10746475 | A | T | A | T | 0.1308 | 0.0037 | 0.0164 | 1.5760E-15 | 0.0225 | 8.6863E-01 | Yes |
| rs11739135 | C | G | C | G | 0.1366 | 0.0002 | 0.0125 | 1.1000E-27 | 0.0173 | 9.9124E-01 | Yes |
| rs1267496 | C | G | C | G | 0.1053 | -0.0400 | 0.0159 | 3.3880E-11 | 0.0208 | 5.4290E-02 | Yes |
| rs1319951 | G | C | G | C | -0.0851 | 0.0776 | 0.0147 | 7.4990E-09 | 0.0196 | 7.8671E-05 | Yes |
| rs1887428 | C | G | C | G | -0.1643 | 0.0286 | 0.0131 | 2.4610E-36 | 0.0178 | 1.0964E-01 | Yes |
| rs1978083 | G | C | G | C | 0.0875 | 0.0099 | 0.0141 | 6.0340E-10 | 0.0191 | 6.0328E-01 | Yes |
| rs2224873 | A | T | A | T | 0.0989 | -0.0038 | 0.0147 | 1.7040E-11 | 0.0210 | 8.5614E-01 | Yes |
| rs34963268 | C | G | C | G | -0.1315 | -0.0897 | 0.0166 | 2.3370E-15 | 0.0225 | 6.8199E-05 | Yes |
| rs35730213 | C | G | C | G | -0.1346 | 0.0068 | 0.0140 | 7.5000E-22 | 0.0193 | 7.2566E-01 | Yes |
| rs4712528 | C | G | C | G | 0.1043 | 0.0027 | 0.0152 | 7.1380E-12 | 0.0204 | 8.9638E-01 | Yes |
| rs77272631 | C | G | C | G | 0.2293 | -0.0595 | 0.0417 | 3.7160E-08 | 0.0562 | 2.8936E-01 | Yes |
| rs77318243 | A | T | A | T | -0.1617 | 0.0497 | 0.0237 | 8.4040E-12 | 0.0353 | 1.5984E-01 | Yes |
| rs8056255 | A | T | A | T | 0.2765 | 0.0378 | 0.0327 | 2.9910E-17 | 0.0431 | 3.8009E-01 | Yes |
| UC |  |  |  |  |  |  |  |  |  |  |  |
| rs10272963 | T | C | T | C | -0.1512 | 0.0193 | 0.0160 | 4.1100E-21 | 0.0171 | 2.5896E-01 | No |
| rs10408351 | A | G | A | G | 0.1548 | 0.0131 | 0.0204 | 2.9200E-14 | 0.0202 | 5.1482E-01 | No |
| rs10737481 | G | T | G | T | 0.2173 | 0.0118 | 0.0159 | 2.5600E-42 | 0.0171 | 4.8838E-01 | No |
| rs10761659 | G | A | G | A | 0.1276 | 0.0023 | 0.0160 | 1.3300E-15 | 0.0170 | 8.9272E-01 | No |
| rs10817678 | A | G | A | G | 0.1332 | -0.0135 | 0.0170 | 4.4200E-15 | 0.0183 | 4.5866E-01 | No |
| rs11209026 | A | G | A | G | -0.4830 | 0.0066 | 0.0358 | 2.0000E-41 | 0.0345 | 8.4809E-01 | No |
| rs1131095 | C | T | C | T | 0.1593 | -0.0040 | 0.0168 | 2.1800E-21 | 0.0182 | 8.2648E-01 | No |
| rs113986290 | T | C | T | C | -0.3066 | 0.0212 | 0.0531 | 7.5900E-09 | 0.0461 | 6.4537E-01 | No |
| rs1157509 | G | A | G | A | 0.1311 | 0.0307 | 0.0217 | 1.5400E-09 | 0.0228 | 1.7684E-01 | No |
| rs11651246 | G | T | G | T | 0.1470 | 0.0214 | 0.0219 | 2.0100E-11 | 0.0256 | 4.0331E-01 | No |
| rs116724447 | A | G | A | G | -0.3390 | 0.0217 | 0.0572 | 3.0100E-09 | 0.0531 | 6.8297E-01 | No |
| rs1265098 | C | T | C | T | -0.1646 | 0.0150 | 0.0203 | 4.7100E-16 | 0.0208 | 4.6877E-01 | No |
| rs12825700 | A | G | A | G | 0.1889 | -0.0147 | 0.0161 | 7.3300E-32 | 0.0177 | 4.0617E-01 | No |
| rs12936409 | T | C | T | C | 0.1365 | -0.0038 | 0.0158 | 5.6200E-18 | 0.0170 | 8.2435E-01 | No |
| rs1317209 | A | G | A | G | 0.1818 | -0.0110 | 0.0203 | 2.9000E-19 | 0.0214 | 6.0529E-01 | No |
| rs13200059 | A | G | A | G | 0.2944 | 0.0131 | 0.0436 | 1.4800E-11 | 0.0543 | 8.0935E-01 | No |
| rs1336900 | A | G | A | G | -0.0887 | -0.0164 | 0.0163 | 4.9500E-08 | 0.0174 | 3.4603E-01 | No |
| rs1359946 | A | G | A | G | 0.1571 | -0.0012 | 0.0202 | 6.5800E-15 | 0.0222 | 9.5864E-01 | No |
| rs137845 | G | A | G | A | 0.1011 | -0.0073 | 0.0158 | 1.5000E-10 | 0.0180 | 6.8666E-01 | No |
| rs138788 | A | G | A | G | 0.0896 | -0.0137 | 0.0162 | 2.9500E-08 | 0.0175 | 4.3482E-01 | No |
| rs1411262 | T | C | T | C | 0.1010 | 0.0163 | 0.0179 | 1.8300E-08 | 0.0198 | 4.1049E-01 | No |
| rs141725002 | A | G | A | G | 0.4885 | -0.0794 | 0.0883 | 3.1600E-08 | 0.0850 | 3.5045E-01 | No |
| rs16830407 | A | G | A | G | 0.1078 | 0.0505 | 0.0166 | 7.6200E-11 | 0.0177 | 4.3470E-03 | No |
| rs16940186 | C | T | C | T | 0.1357 | 0.0063 | 0.0214 | 2.1900E-10 | 0.0215 | 7.6968E-01 | No |
| rs17190351 | A | G | A | G | 0.4301 | 0.0948 | 0.0561 | 1.7900E-14 | 0.0603 | 1.1596E-01 | No |
| rs17202899 | C | T | C | T | 0.2211 | -0.0054 | 0.0312 | 1.4300E-12 | 0.0251 | 8.3079E-01 | No |
| rs1736161 | A | G | A | G | -0.1227 | 0.0074 | 0.0161 | 2.2200E-14 | 0.0173 | 6.7021E-01 | No |
| rs17656349 | T | C | T | C | 0.0900 | 0.0021 | 0.0159 | 1.5400E-08 | 0.0172 | 9.0344E-01 | No |
| rs17715902 | A | G | A | G | 0.0974 | 0.0290 | 0.0166 | 4.6200E-09 | 0.0180 | 1.0648E-01 | No |
| rs1846190 | A | G | A | G | -0.2267 | 0.0533 | 0.0190 | 1.1600E-32 | 0.0184 | 3.7882E-03 | No |
| rs2045241 | A | G | A | G | -0.1063 | 0.0101 | 0.0169 | 2.8300E-10 | 0.0181 | 5.7757E-01 | No |
| rs2212434 | T | C | T | C | 0.1252 | 0.0069 | 0.0159 | 2.8000E-15 | 0.0170 | 6.8217E-01 | No |
| rs2294633 | T | C | T | C | -0.0985 | 0.0010 | 0.0177 | 2.7900E-08 | 0.0191 | 9.5777E-01 | No |
| rs2301989 | A | G | A | G | -0.1294 | -0.0084 | 0.0161 | 1.0800E-15 | 0.0174 | 6.2851E-01 | No |
| rs2816980 | T | G | T | G | 0.1941 | 0.0247 | 0.0260 | 9.1800E-14 | 0.0270 | 3.6032E-01 | No |
| rs2836881 | T | G | T | G | -0.2217 | -0.0135 | 0.0186 | 1.1100E-32 | 0.0192 | 4.8170E-01 | No |
| rs2838517 | C | T | C | T | -0.1177 | 0.0161 | 0.0160 | 1.7800E-13 | 0.0175 | 3.5697E-01 | No |
| rs3024493 | A | C | A | C | 0.2100 | 0.0209 | 0.0209 | 7.4600E-24 | 0.0237 | 3.7642E-01 | No |
| rs34920465 | G | A | G | A | -0.1708 | -0.0897 | 0.0213 | 9.0100E-16 | 0.0224 | 6.3900E-05 | No |
| rs3812565 | C | T | C | T | 0.1335 | 0.0057 | 0.0160 | 6.5000E-17 | 0.0178 | 7.4920E-01 | No |
| rs3820328 | G | A | G | A | -0.1662 | -0.0088 | 0.0164 | 3.6600E-24 | 0.0177 | 6.1854E-01 | No |
| rs3823377 | A | C | A | C | 0.1482 | -0.0090 | 0.0196 | 3.6000E-14 | 0.0185 | 6.2490E-01 | No |
| rs41291790 | A | G | A | G | 0.9494 | 0.1045 | 0.0806 | 5.3300E-32 | 0.1135 | 3.5730E-01 | No |
| rs45593732 | T | C | T | C | 0.4903 | -0.1189 | 0.0523 | 6.3500E-21 | 0.0639 | 6.2933E-02 | No |
| rs4676408 | A | G | A | G | 0.1433 | 0.0135 | 0.0167 | 1.1900E-17 | 0.0181 | 4.5601E-01 | No |
| rs4728142 | A | G | A | G | 0.0995 | -0.0287 | 0.0158 | 3.2300E-10 | 0.0172 | 9.5218E-02 | No |
| rs4845604 | A | G | A | G | -0.1608 | -0.0091 | 0.0239 | 1.5700E-11 | 0.0242 | 7.0776E-01 | No |
| rs4993442 | T | G | T | G | -0.0988 | -0.0022 | 0.0179 | 3.5400E-08 | 0.0190 | 9.0683E-01 | No |
| rs56062135 | T | C | T | C | 0.1078 | 0.0197 | 0.0184 | 4.6600E-09 | 0.0203 | 3.3088E-01 | No |
| rs6017342 | C | A | C | A | 0.1944 | -0.0365 | 0.0170 | 3.9500E-30 | 0.0184 | 4.6756E-02 | No |
| rs6062496 | A | G | A | G | 0.1359 | -0.0458 | 0.0163 | 8.9700E-17 | 0.0173 | 8.1964E-03 | No |
| rs62180181 | T | C | T | C | 0.1226 | 0.0273 | 0.0171 | 8.0800E-13 | 0.0195 | 1.6183E-01 | No |
| rs67111717 | G | A | G | A | 0.0944 | 0.0087 | 0.0171 | 3.2700E-08 | 0.0177 | 6.2246E-01 | No |
| rs6889364 | A | G | A | G | 0.1318 | -0.0286 | 0.0228 | 7.8700E-09 | 0.0256 | 2.6300E-01 | No |
| rs6915986 | C | T | C | T | 0.4040 | 0.0028 | 0.0616 | 5.4500E-11 | 0.0452 | 9.4986E-01 | No |
| rs6933404 | C | T | C | T | 0.1486 | 0.0108 | 0.0188 | 2.6900E-15 | 0.0215 | 6.1694E-01 | No |
| rs72704802 | T | C | T | C | -0.1223 | -0.0008 | 0.0206 | 2.8900E-09 | 0.0229 | 9.7164E-01 | No |
| rs7523335 | A | G | A | G | -0.1389 | 0.0007 | 0.0210 | 3.4200E-11 | 0.0225 | 9.7496E-01 | No |
| rs755374 | T | C | T | C | 0.1714 | 0.0168 | 0.0171 | 9.7300E-24 | 0.0195 | 3.8723E-01 | No |
| rs7554511 | A | C | A | C | -0.1448 | 0.0060 | 0.0178 | 4.2700E-16 | 0.0192 | 7.5516E-01 | No |
| rs7608697 | C | A | C | A | 0.1597 | 0.0068 | 0.0161 | 3.0300E-23 | 0.0173 | 6.9484E-01 | No |
| rs78064630 | A | G | A | G | 0.1759 | 0.0117 | 0.0308 | 1.0800E-08 | 0.0316 | 7.1173E-01 | No |
| rs79051659 | A | G | A | G | 0.1605 | 0.0098 | 0.0264 | 1.3000E-09 | 0.0251 | 6.9550E-01 | No |
| rs7911117 | G | T | G | T | -0.1342 | 0.0275 | 0.0239 | 1.8400E-08 | 0.0243 | 2.5919E-01 | No |
| rs7911680 | C | A | C | A | -0.1525 | 0.0401 | 0.0159 | 6.7100E-22 | 0.0170 | 1.8082E-02 | No |
| rs8073117 | A | G | A | G | -0.1548 | 0.0192 | 0.0225 | 6.4000E-12 | 0.0233 | 4.0906E-01 | No |
| rs9263719 | T | C | T | C | -0.1692 | -0.0277 | 0.0269 | 3.0900E-10 | 0.0249 | 2.6416E-01 | No |
| rs9271176 | G | A | G | A | -0.3495 | 0.0239 | 0.0173 | 4.2000E-91 | 0.0182 | 1.8968E-01 | No |
| rs9611131 | C | T | C | T | -0.1494 | 0.0290 | 0.0227 | 5.1100E-11 | 0.0235 | 2.1872E-01 | No |
| rs11645239 | G | C | G | C | -0.1174 | -0.0035 | 0.0200 | 4.1400E-09 | 0.0207 | 8.6480E-01 | Yes |
| rs1811711 | G | C | G | C | -0.1299 | 0.0465 | 0.0223 | 6.0900E-09 | 0.0244 | 5.6392E-02 | Yes |
| rs1887428 | C | G | C | G | -0.1670 | 0.0286 | 0.0166 | 9.6500E-24 | 0.0178 | 1.0964E-01 | Yes |
| rs6658353 | C | G | C | G | -0.1569 | 0.0113 | 0.0160 | 1.1700E-22 | 0.0171 | 5.1098E-01 | Yes |
| rs872956 | A | T | A | T | -0.1378 | 0.0110 | 0.0210 | 5.2400E-11 | 0.0220 | 6.1905E-01 | Yes |
| CD |  |  |  |  |  |  |  |  |  |  |  |
| rs10055349 | A | G | A | G | 0.1734 | -0.0149 | 0.0190 | 5.5900E-20 | 0.0209 | 4.7484E-01 | No |
| rs10114470 | C | T | C | T | 0.1687 | -0.0005 | 0.0177 | 1.7600E-21 | 0.0187 | 9.7884E-01 | No |
| rs1012636 | T | G | T | G | 0.1291 | -0.0015 | 0.0198 | 7.0100E-11 | 0.0207 | 9.4208E-01 | No |
| rs10822050 | C | T | C | T | 0.1827 | 0.0336 | 0.0162 | 2.3500E-29 | 0.0173 | 5.3001E-02 | No |
| rs10884966 | A | G | A | G | 0.1131 | -0.0417 | 0.0171 | 4.1300E-11 | 0.0182 | 2.1811E-02 | No |
| rs11236797 | A | C | A | C | 0.1760 | 0.0092 | 0.0161 | 8.5100E-28 | 0.0170 | 5.8676E-01 | No |
| rs114802258 | T | C | T | C | -0.2245 | 0.0145 | 0.0384 | 5.1100E-09 | 0.0421 | 7.2987E-01 | No |
| rs1148246 | T | C | T | C | -0.1323 | -0.0187 | 0.0167 | 2.0900E-15 | 0.0179 | 2.9582E-01 | No |
| rs1157509 | G | A | G | A | 0.1519 | 0.0307 | 0.0224 | 1.2600E-11 | 0.0228 | 1.7684E-01 | No |
| rs11677002 | C | T | C | T | -0.1124 | 0.0109 | 0.0163 | 4.5700E-12 | 0.0175 | 5.3306E-01 | No |
| rs11683692 | C | T | C | T | -0.2144 | -0.0014 | 0.0380 | 1.7500E-08 | 0.0385 | 9.7030E-01 | No |
| rs11965964 | T | C | T | C | 0.3044 | -0.0448 | 0.0529 | 8.8300E-09 | 0.0519 | 3.8808E-01 | No |
| rs12041056 | T | C | T | C | 0.1284 | -0.0171 | 0.0163 | 3.7500E-15 | 0.0174 | 3.2572E-01 | No |
| rs1250573 | A | G | A | G | -0.1522 | 0.0904 | 0.0179 | 1.9200E-17 | 0.0190 | 2.0600E-06 | No |
| rs1260326 | C | T | C | T | -0.1053 | 0.0337 | 0.0161 | 6.3200E-11 | 0.0175 | 5.3688E-02 | No |
| rs12936409 | T | C | T | C | 0.1426 | -0.0038 | 0.0160 | 4.3100E-19 | 0.0170 | 8.2435E-01 | No |
| rs1297264 | G | A | G | A | -0.1769 | 0.0097 | 0.0163 | 1.5900E-27 | 0.0172 | 5.7181E-01 | No |
| rs13107325 | T | C | T | C | 0.2006 | 0.0253 | 0.0284 | 1.6700E-12 | 0.0318 | 4.2624E-01 | No |
| rs1321859 | T | C | T | C | -0.1049 | 0.0110 | 0.0172 | 1.1800E-09 | 0.0190 | 5.6394E-01 | No |
| rs1373904 | G | A | G | A | 0.1410 | -0.0340 | 0.0189 | 9.1100E-14 | 0.0197 | 8.4496E-02 | No |
| rs137976175 | A | G | A | G | -0.2564 | 0.0106 | 0.0372 | 5.5900E-12 | 0.0372 | 7.7507E-01 | No |
| rs142770866 | A | G | A | G | 0.1753 | 0.0082 | 0.0292 | 1.9900E-09 | 0.0305 | 7.8750E-01 | No |
| rs144309607 | T | C | T | C | -0.3712 | 0.0753 | 0.0470 | 2.6900E-15 | 0.0448 | 9.2791E-02 | No |
| rs145126485 | C | A | C | A | 0.5236 | 0.0144 | 0.0431 | 6.4000E-34 | 0.0494 | 7.7041E-01 | No |
| rs145568234 | G | T | G | T | 0.8602 | -0.0398 | 0.0633 | 4.3100E-42 | 0.0754 | 5.9808E-01 | No |
| rs149399409 | A | G | A | G | 0.2584 | -0.0413 | 0.0398 | 8.3900E-11 | 0.0493 | 4.0219E-01 | No |
| rs1583792 | T | C | T | C | -0.0882 | -0.0317 | 0.0160 | 3.2600E-08 | 0.0170 | 6.2681E-02 | No |
| rs1775448 | G | A | G | A | -0.1220 | -0.0009 | 0.0170 | 6.7800E-13 | 0.0186 | 9.6270E-01 | No |
| rs181826 | A | C | A | C | 0.1162 | -0.0314 | 0.0167 | 3.2500E-12 | 0.0177 | 7.5839E-02 | No |
| rs1870148 | A | G | A | G | 0.1351 | -0.0139 | 0.0206 | 5.4400E-11 | 0.0220 | 5.2760E-01 | No |
| rs194746 | T | C | T | C | 0.0975 | -0.0010 | 0.0161 | 1.2400E-09 | 0.0171 | 9.5536E-01 | No |
| rs1990684 | T | C | T | C | -0.1556 | 0.0126 | 0.0280 | 2.7300E-08 | 0.0286 | 6.5970E-01 | No |
| rs2002695 | G | A | G | A | -0.1293 | -0.0090 | 0.0189 | 8.3100E-12 | 0.0199 | 6.5016E-01 | No |
| rs2021511 | T | C | T | C | -0.1082 | -0.0004 | 0.0182 | 2.6300E-09 | 0.0188 | 9.8401E-01 | No |
| rs2076756 | G | A | G | A | 0.3850 | 0.0019 | 0.0174 | 1.8000E-108 | 0.0202 | 9.2559E-01 | No |
| rs2110735 | G | A | G | A | -0.1372 | 0.0329 | 0.0185 | 1.2000E-13 | 0.0198 | 9.6799E-02 | No |
| rs212408 | T | G | T | G | -0.1136 | -0.0286 | 0.0167 | 9.1200E-12 | 0.0180 | 1.1254E-01 | No |
| rs2143178 | C | T | C | T | -0.2087 | 0.0194 | 0.0223 | 6.8400E-21 | 0.0225 | 3.8965E-01 | No |
| rs2188962 | T | C | T | C | 0.2004 | -0.0095 | 0.0160 | 5.5900E-36 | 0.0172 | 5.8145E-01 | No |
| rs2240069 | G | A | G | A | -0.1568 | -0.0185 | 0.0252 | 4.7300E-10 | 0.0239 | 4.3802E-01 | No |
| rs2284553 | G | A | G | A | 0.1277 | -0.0149 | 0.0165 | 1.1400E-14 | 0.0179 | 4.0675E-01 | No |
| rs2476601 | G | A | G | A | 0.2312 | -0.0159 | 0.0286 | 6.4400E-16 | 0.0304 | 6.0048E-01 | No |
| rs2838517 | C | T | C | T | -0.1456 | 0.0161 | 0.0162 | 2.0300E-19 | 0.0175 | 3.5697E-01 | No |
| rs28999107 | T | G | T | G | 0.1083 | -0.0259 | 0.0178 | 1.0600E-09 | 0.0186 | 1.6240E-01 | No |
| rs2948542 | G | A | G | A | 0.1016 | 0.0036 | 0.0163 | 5.1500E-10 | 0.0171 | 8.3206E-01 | No |
| rs3091315 | G | A | G | A | -0.1579 | -0.0096 | 0.0182 | 3.7600E-18 | 0.0189 | 6.1320E-01 | No |
| rs3122605 | A | G | A | G | -0.1748 | -0.0139 | 0.0227 | 1.2400E-14 | 0.0251 | 5.8059E-01 | No |
| rs34635748 | T | C | T | C | 0.4794 | -0.0194 | 0.0504 | 1.9500E-21 | 0.0495 | 6.9547E-01 | No |
| rs34687326 | A | G | A | G | -0.1649 | 0.0047 | 0.0288 | 1.0600E-08 | 0.0303 | 8.7718E-01 | No |
| rs35171809 | G | A | G | A | 0.1566 | 0.0256 | 0.0159 | 9.0700E-23 | 0.0170 | 1.3116E-01 | No |
| rs3761158 | A | G | A | G | -0.1098 | 0.0225 | 0.0165 | 2.6500E-11 | 0.0174 | 1.9717E-01 | No |
| rs3812609 | T | C | T | C | -0.1443 | -0.0007 | 0.0247 | 4.9600E-09 | 0.0278 | 9.8097E-01 | No |
| rs3816234 | A | G | A | G | 0.2704 | -0.0069 | 0.0162 | 1.5100E-62 | 0.0171 | 6.8657E-01 | No |
| rs3850378 | C | T | C | T | 0.1990 | -0.0013 | 0.0267 | 8.3100E-14 | 0.0304 | 9.6541E-01 | No |
| rs401775 | C | T | C | T | 0.2023 | 0.0118 | 0.0203 | 2.1700E-23 | 0.0219 | 5.9028E-01 | No |
| rs4077515 | T | C | T | C | 0.1848 | 0.0066 | 0.0162 | 3.1500E-30 | 0.0176 | 7.0818E-01 | No |
| rs42861 | G | A | G | A | 0.1243 | 0.0107 | 0.0167 | 8.8700E-14 | 0.0178 | 5.4721E-01 | No |
| rs4343432 | G | A | G | A | 0.1123 | 0.0172 | 0.0162 | 3.5000E-12 | 0.0171 | 3.1486E-01 | No |
| rs4380956 | A | G | A | G | 0.1320 | 0.0145 | 0.0165 | 1.1500E-15 | 0.0176 | 4.0836E-01 | No |
| rs4486887 | T | C | T | C | -0.1686 | -0.0025 | 0.0172 | 1.3700E-22 | 0.0190 | 8.9544E-01 | No |
| rs4655709 | A | G | A | G | 0.1224 | -0.0268 | 0.0183 | 2.4600E-11 | 0.0190 | 1.5846E-01 | No |
| rs4807570 | A | G | A | G | 0.1811 | -0.0110 | 0.0193 | 6.0300E-21 | 0.0214 | 6.0768E-01 | No |
| rs4807633 | T | C | T | C | 0.1301 | -0.0315 | 0.0189 | 5.6400E-12 | 0.0214 | 1.4017E-01 | No |
| rs4821544 | C | T | C | T | 0.0966 | -0.0315 | 0.0171 | 1.7600E-08 | 0.0181 | 8.2678E-02 | No |
| rs492602 | G | A | G | A | 0.1084 | 0.0085 | 0.0162 | 2.3300E-11 | 0.0170 | 6.1811E-01 | No |
| rs55946629 | A | C | A | C | 0.1755 | -0.0619 | 0.0231 | 2.8500E-14 | 0.0260 | 1.7230E-02 | No |
| rs56116661 | T | C | T | C | -0.1312 | 0.0421 | 0.0212 | 5.6700E-10 | 0.0216 | 5.1247E-02 | No |
| rs5754100 | C | T | C | T | 0.1687 | 0.0172 | 0.0206 | 3.0200E-16 | 0.0219 | 4.3208E-01 | No |
| rs59805578 | C | T | C | T | -0.2674 | -0.0179 | 0.0340 | 3.9200E-15 | 0.0364 | 6.2270E-01 | No |
| rs59926756 | A | G | A | G | 0.1062 | 0.0246 | 0.0176 | 1.7400E-09 | 0.0192 | 2.0064E-01 | No |
| rs6062496 | A | G | A | G | 0.1223 | -0.0458 | 0.0167 | 2.6200E-13 | 0.0173 | 8.1964E-03 | No |
| rs61839660 | T | C | T | C | 0.1468 | -0.0437 | 0.0261 | 1.9800E-08 | 0.0307 | 1.5453E-01 | No |
| rs62126620 | A | G | A | G | 0.1440 | 0.0090 | 0.0201 | 8.6100E-13 | 0.0208 | 6.6415E-01 | No |
| rs62228374 | A | G | A | G | 0.3164 | 0.0861 | 0.0557 | 1.3600E-08 | 0.0592 | 1.4616E-01 | No |
| rs62324212 | A | C | A | C | 0.1060 | -0.0289 | 0.0163 | 8.0200E-11 | 0.0175 | 9.9646E-02 | No |
| rs6416647 | C | T | C | T | 0.1007 | -0.0072 | 0.0178 | 1.4600E-08 | 0.0193 | 7.1063E-01 | No |
| rs6451494 | C | T | C | T | 0.2605 | -0.0616 | 0.0166 | 8.2600E-56 | 0.0174 | 4.0879E-04 | No |
| rs6584282 | G | A | G | A | -0.1658 | 0.0420 | 0.0160 | 3.4400E-25 | 0.0170 | 1.3215E-02 | No |
| rs6704109 | T | C | T | C | 0.1748 | -0.0860 | 0.0181 | 5.1000E-22 | 0.0197 | 1.2500E-05 | No |
| rs6740847 | G | A | G | A | -0.1040 | -0.0031 | 0.0161 | 9.7200E-11 | 0.0170 | 8.5494E-01 | No |
| rs6808936 | G | A | G | A | 0.0904 | 0.0367 | 0.0161 | 1.9300E-08 | 0.0172 | 3.2868E-02 | No |
| rs72743461 | A | C | A | C | 0.1684 | 0.0203 | 0.0187 | 2.2600E-19 | 0.0202 | 3.1483E-01 | No |
| rs72748445 | A | C | A | C | -0.1369 | 0.0974 | 0.0181 | 4.3100E-14 | 0.0188 | 2.1700E-07 | No |
| rs72798422 | C | T | C | T | 0.5495 | 0.0433 | 0.0382 | 6.0500E-47 | 0.0431 | 3.1517E-01 | No |
| rs73243877 | G | A | G | A | 0.1164 | 0.0263 | 0.0212 | 4.1200E-08 | 0.0225 | 2.4216E-01 | No |
| rs73516754 | C | A | C | A | 0.1423 | 0.0038 | 0.0169 | 4.0400E-17 | 0.0183 | 8.3788E-01 | No |
| rs744166 | G | A | G | A | -0.1142 | 0.0212 | 0.0162 | 1.8000E-12 | 0.0172 | 2.1767E-01 | No |
| rs7517847 | G | T | G | T | -0.3447 | 0.0165 | 0.0165 | 5.8400E-97 | 0.0174 | 3.4231E-01 | No |
| rs755374 | T | C | T | C | 0.1969 | 0.0168 | 0.0174 | 1.3800E-29 | 0.0195 | 3.8723E-01 | No |
| rs7563433 | C | T | C | T | 0.1525 | 0.0216 | 0.0200 | 2.1400E-14 | 0.0220 | 3.2606E-01 | No |
| rs7608697 | C | A | C | A | 0.1229 | 0.0068 | 0.0163 | 4.0300E-14 | 0.0173 | 6.9484E-01 | No |
| rs76532080 | T | C | T | C | 0.2939 | 0.0409 | 0.0352 | 6.9600E-17 | 0.0424 | 3.3426E-01 | No |
| rs79832570 | C | T | C | T | 0.2234 | -0.0291 | 0.0344 | 8.9000E-11 | 0.0339 | 3.9061E-01 | No |
| rs80262450 | A | G | A | G | 0.2268 | 0.0212 | 0.0244 | 1.3400E-20 | 0.0277 | 4.4466E-01 | No |
| rs938650 | A | G | A | G | -0.1747 | -0.0312 | 0.0247 | 1.6500E-12 | 0.0252 | 2.1599E-01 | No |
| rs9482770 | C | T | C | T | 0.0987 | 0.0349 | 0.0162 | 1.0100E-09 | 0.0172 | 4.2773E-02 | No |
| rs9501109 | G | A | G | A | 0.1381 | 0.0288 | 0.0209 | 3.8400E-11 | 0.0199 | 1.4851E-01 | No |
| rs9637870 | A | G | A | G | 0.2558 | -0.0411 | 0.0275 | 1.3300E-20 | 0.0277 | 1.3863E-01 | No |
| rs9656588 | C | T | C | T | 0.1183 | -0.0110 | 0.0173 | 8.7300E-12 | 0.0184 | 5.4769E-01 | No |
| rs9836291 | A | G | A | G | 0.1722 | -0.0007 | 0.0170 | 3.7700E-24 | 0.0182 | 9.7028E-01 | No |
| rs10052709 | G | C | G | C | -0.1410 | 0.0384 | 0.0242 | 5.7600E-09 | 0.0240 | 1.1013E-01 | Yes |
| rs151175749 | G | C | G | C | 0.2483 | -0.0004 | 0.0428 | 6.7300E-09 | 0.0528 | 9.9346E-01 | Yes |
| rs1887428 | C | G | C | G | -0.1660 | 0.0286 | 0.0169 | 8.5400E-23 | 0.0178 | 1.0964E-01 | Yes |
| rs2581828 | G | C | G | C | -0.0941 | 0.0080 | 0.0162 | 6.4600E-09 | 0.0174 | 6.4483E-01 | Yes |
| rs2675670 | C | G | C | G | 0.1074 | -0.0305 | 0.0161 | 2.9000E-11 | 0.0171 | 7.4791E-02 | Yes |
| rs35730213 | C | G | C | G | -0.1166 | 0.0068 | 0.0181 | 1.1700E-10 | 0.0193 | 7.2566E-01 | Yes |
| rs59145923 | C | G | C | G | -0.1717 | 0.0156 | 0.0309 | 2.7200E-08 | 0.0335 | 6.4108E-01 | Yes |
| rs7198678 | T | A | T | A | -0.1398 | -0.0133 | 0.0229 | 1.0800E-09 | 0.0243 | 5.8472E-01 | Yes |
| rs7206852 | A | T | A | T | -0.1287 | 0.0489 | 0.0223 | 7.7100E-09 | 0.0235 | 3.7299E-02 | Yes |
| rs7753014 | G | C | G | C | -0.0989 | 0.0150 | 0.0163 | 1.3900E-09 | 0.0171 | 3.8066E-01 | Yes |
| rs9276772 | G | C | G | C | -0.1857 | 0.0493 | 0.0269 | 5.1500E-12 | 0.0297 | 9.7544E-02 | Yes |

Abbreviations: CRC, colorectal cancer, IBD, inflammatory bowel disease, UC, ulcerative colitis, CD, Crohn's disease.

## Supplementary Table 3. Sources of GWAS summary statistics.

| **Mapped Trait** | **Trait type** | **Source** | **Reference** | **GWAS ID/prefix** | **Sample sizes** | **Cases/Controls** |
| --- | --- | --- | --- | --- | --- | --- |
| Haemoglobin concentration | Quantitative | GWAS Catalog | PMID: 34017140 | GCST90013978 | 396,624 | Not applicable |
| C-reactive protein levels | Quantitative | GWAS Catalog | PMID: 35459240 | GCST90029070 | 575,531 | Not applicable |
| Neutrophill count | Quantitative | GWAS Catalog | PMID: 34017140 | GCST90013984 | 395,949 | Not applicable |
| Lymphocyte cell count | Quantitative | Blood Cell Consortium | <https://gwas.mrcieu.ac.uk/datasets/ieu-b-32/> | ieu-b-32 | 563,946 | Not applicable |
| Inflammatory bowel disease | Binary | GWAS Catalog | PMID: 28067908 | GCST004131 | 59,957 | 25,042/34,915 |
| Crohn's disease | Binary | GWAS Catalog | PMID: 28067908 | GCST004132 | 40,266 | 12,194/28,072 |
| Ulcerative colitis | Binary | GWAS Catalog | PMID: 28067908 | GCST004133 | 45,975 | 12,366/33,609 |
| Colorectal cancer | Binary | GWAS Catalog | PMID: 30510241 | GCST012879 | 32,072 | 19,948/12,124 |

## Supplementary Table 4. Variance inflation factor (VIF) results for multivariate model incorporating different disease severity associated laboratory biomarkers.

| Biomarker | CRP | Hgb | Platelet | Albumin | NLR | PLR | Neutrophil | SII |
| --- | --- | --- | --- | --- | --- | --- | --- | --- |
| VIF | 1.1803 | 1.1604 | 7.0972 | 1.1283 | 21.7049 | 13.1034 | 8.6251 | 26.4817 |
|  | 1.1765 | 1.1584 | 5.1300 | 1.1283 | 13.4621 | 11.1163 | 7.6389 | Removed |
|  | 1.1674 | 1.1475 | 1.6106 | 1.1267 | 2.0613 | 2.6059 | Removed | Removed |

Abbreviations: VIF, variance inflation factor, CRP, C-reactive protein, Hgb, haemoglobin, NLR, neutrophil-lymphocyte ratio, PLR, platelet-lymphocyte ratio, SII, systemic immune-inflammation index.

## Supplementary Table 5. Associations between diagnosis for any IBD, UC, CD and risk of CRC by sex.

|  | Person years | n CRC/N total (% CRC) | Model 1 HR (95%CIs)^1^ | *P-value* | Model 2 HR (95%CIs)^2^ | *P-value* |
| --- | --- | --- | --- | --- | --- | --- |
| Male |  |  |  |  |  |  |
| No IBD | 2358085.01 | 3024/204337 (1.48%) | Ref. |  | Ref. |  |
| Any IBD | 27655.69 | 56/2433 (2.30%) | 1.51 (1.16,1.97) | 0.002 | 1.71 (1.31,2.24) | <0.001 |
| UC | 19075.80 | 42/1676 (2.51%) | 1.62 (1.19,2.19) | 0.002 | 1.87 (1.37,2.53) | <0.001 |
| CD | 8579.90 | 14/757 (1.85%) | 1.26 (0.74,2.12) | 0.393 | 1.36 (0.79,2.35) | 0.267 |
| Female |  |  |  |  |  |  |
| No IBD | 2723673.73 | 2220/231993 (0.96%) | Ref. |  | Ref. |  |
| Any IBD | 29773.22 | 42/2558 (1.64%) | 1.66 (1.22,2.25) | 0.001 | 1.87 (1.37,2.55) | <0.001 |
| UC | 19004.02 | 31/1625 (1.91%) | 1.88 (1.32,2.68) | <0.001 | 2.12 (1.49,3.04) | <0.001 |
| CD | 10769.20 | 11/933 (1.18%) | 1.25 (0.69,2.26) | 0.459 | 1.41 (0.78,2.55) | 0.259 |

^1^ Model 1: Estimated from the Cox regression model with age as the underlying time scale, adjusted for sex, age at recruitment, race (white, asian, african, mixed background, unknown) in UK Biobank.

^2^ Model 2: Further adjusted for Townsend deprivation index (continuous), region of the recruitment assessment center, educational level (college/university degree, non-college/university degree, unknown), CRC screening history (no, yes, unknown), family history of CRC (no, yes, unknown), standard polygenic risk score for CRC (continuous), healthy lifestyle index and IBD disease course in UK Biobank.

Abbreviations: CI, confidence interval, HR, hazard ratio, CRC, colorectal cancer, IBD, inflammatory bowel disease, UC, ulcerative colitis, CD, Crohn's disease.

## Supplementary Table 6. Sensitivity analyses of associations between diagnosis for any IBD, UC, CD and risk of CRC.

| Exposure | Person years | n CRC/N total (% CRC) | Model 1 HR (95%CIs)^1^ | *P-value* | Model 2 HR (95%CIs)^2^ | *P-value* |
| --- | --- | --- | --- | --- | --- | --- |
| Excluding CRC cases within the first 2 years of follow-up | | | | | | |
| No IBD | 5080991.52 | 4505/435591 (1.03%) | Ref. |  | Ref. |  |
| Any IBD | 57409.82 | 82/4975 (1.65%) | 1.53 (1.23,1.91) | <0.001 | 1.73 (1.38,2.15) | <0.001 |
| UC | 38067.78 | 63/3291 (1.91%) | 1.73 (1.35,2.22) | <0.001 | 1.97 (1.54,2.54) | <0.001 |
| CD | 19342.03 | 19/1684 (1.26%) | 1.11 (0.71,1.75) | 0.640 | 1.21 (0.76,1.92) | 0.430 |
| Excluding IBD cases diagnosed after baseline | | | | | | |
| No IBD | 5048537.32 | 5170/433464 (1.19%) | Ref. |  | Ref. |  |
| Any IBD | 57428.92 | 98/4991 (1.96%) | 1.58 (1.29,1.93) | <0.001 | 1.79 (1.46,2.19) | <0.001 |
| UC | 38079.82 | 73/3301 (2.21%) | 1.73 (1.37,2.18) | <0.001 | 1.98 (1.57,2.50) | <0.001 |
| CD | 19349.10 | 25/1690 (1.48%) | 1.26 (0.85,1.87) | 0.243 | 1.39 (0.93,2.08) | 0.107 |
| Excluding indeterminate colitis | | | | | | |
| No IBD | 5081758.73 | 5244/436330 (1.20%) | Ref. |  | Ref. |  |
| Any IBD | 49185.85 | 79/4276 (1.85%) | 1.47 (1.18,1.84) | <0.001 | 1.67 (1.34,2.09) | <0.001 |
| UC | 34535.70 | 65/2994 (2.17%) | 1.67 (1.31,2.13) | <0.001 | 1.90 (1.49,2.43) | <0.001 |
| CD | 14650.15 | 14/1282 (1.09%) | 0.95 (0.56,1.60) | 0.833 | 1.07 (0.63,1.81) | 0.794 |

^1^ Model 1: Estimated from the Cox regression model with age as the underlying time scale, adjusted for sex, age at recruitment, race (white, asian, african, mixed background, unknown) in UK Biobank.

^2^ Model 2: Further adjusted for Townsend deprivation index (continuous), region of the recruitment assessment center, educational level (college/university degree, non-college/university degree, unknown), CRC screening history (no, yes, unknown), family history of CRC (no, yes, unknown), standard polygenic risk score for CRC (continuous), healthy lifestyle index and IBD disease course in UK Biobank.

Abbreviations: CI, confidence interval, HR, hazard ratio, CRC, colorectal cancer, IBD, inflammatory bowel disease, UC, ulcerative colitis, CD, Crohn's disease.

## Supplementary Table 7. Genetic correlation between the liability to IBD, UC, and CD and CRC.

| Trait 1 | Trait 2 | Rg^1^ (95% CIs) | *P* |
| --- | --- | --- | --- |
| IBD | CRC | -0.1145 (-0.2934, 0.0644) | 0.2097 |
| CD | CRC | -0.1176 (-0.3118, 0.0766) | 0.2355 |
| UC | CRC | -0.0822 (-0.2874, 0.1230) | 0.4322 |

^1^ Rg: Genetic correlation coefficients.

Abbreviations: CI, confidence interval; CRC, colorectal cancer; IBD, inflammatory bowel disease; UC, ulcerative colitis; CD, Crohn's disease.

## Supplementary Table 8. F statistic and power calculation for Mendelian randomization analyses for IBD, UC, CD in relation to CRC.

| **Trait** | **Nsnps** | **Sample size** | **No. cases** | **No. controls** | **R^2^** | **F statistic** | **Power^1^** |
| --- | --- | --- | --- | --- | --- | --- | --- |
| IBD | 122 | 59,957 | 25,042 | 34,915 | 0.1516 | 11.54 | 0.83 |
| UC | 69 | 45,975 | 12,366 | 33,609 | 0.1299 | 11.42 | 0.41 |
| CD | 100 | 40,266 | 12,194 | 28,072 | 0.1987 | 16.52 | 0.84 |

^1^ Power calculated using <https://shiny.cnsgenomics.com/mRnd/>

Abbreviations: IBD, inflammatory bowel disease, UC, ulcerative colitis, CD, Crohn's disease

## Supplementary Table 9. Mediation analysis of the disease severity associated laboratory biomarkers for the association between IBD and CRC.

| **Mediator** | **TE (95%CI) ×10^-7^** | **DE (95%CI) ×10^-7^** | **IE (95%CI) ×10^-7^** | **Proportion mediated** |
| --- | --- | --- | --- | --- |
| CRP | 17.97 (11.20, 24.74) | 16.10 (9.37, 22.82) | 1.87 (1.06, 2.68) | 10.41% |
| Platelet | 17.89 (11.21, 24.56) | 18.20 (11.61, 24.78) | -0.31 (-1.39, 0.78) | - |
| Neutrophil | 18.49 (11.77, 25.21) | 17.70 (11.10, 24.30) | 0.79 (-0.32, 1.91) | - |
| PLR | 17.85 (10.85, 24.85) | 17.90 (11.00, 24.79) | -0.05 (-1.23, 1.14) | - |
| NLR | 18.66 (11.82, 25.49) | 16.80 (10.00, 23.60) | 1.86 (1.14, 2.57) | 9.97% |
| SII | 17.97 (10.92, 25.02) | 16.50 (9.66, 23.30) | 1.47 (-0.25, 3.19) | - |
| Hgb | 18.04 (11.47, 24.61) | 18.50 (11.93, 25.06) | -0.46 (-0.58, -0.35) | 2.55% |
| Albumin | 17.02 (10.51, 23.52) | 16.20 (9.79, 22.60) | 0.82 (-0.29, 1.93) | - |

TE, DE, and IE correspond to total, natural direct, and natural indirect effect, respectively.

Abbreviations: CRP, C-reactive protein; Hgb, haemoglobin; NLR, Neutrophil-lymphocyte ratio; PLR, platelet-lymphocyte ratio, SII, systemic immune-inflammation index.

## Supplementary Table 10. Association between IBD and GM using inverse variance weighted method.

| **Exposure** | **Outcome** | | | **SNPs** | **Beta** | **Standard error** | ***P*** |
| --- | --- | --- | --- | --- | --- | --- | --- |
|  | **Taxon level** | **Trait** | **MiBioGen ID** |  |  |  |  |
| Inflammatory bowel disease | genus | *Eubacterium ruminantium group* | id.11340 | 13 | -7.362 | 3.620 | 0.042 |
|  | genus | *Barnesiella* | id.944 | 13 | 6.660 | 3.283 | 0.042 |
|  | genus | *Olsenella* | id.822 | 13 | 10.550 | 5.098 | 0.039 |
|  | genus | *Phascolarctobacterium* | id.2168 | 13 | -5.981 | 3.022 | 0.048 |
|  | genus | *Rikenellaceae rc9 gut group* | id.11191 | 12 | 11.595 | 5.833 | 0.047 |
|  | genus | *Ruminococcaceae UCG 003* | id.11361 | 13 | -6.730 | 2.650 | 0.011 |
|  | genus | *Ruminococcus2* | id.11374 | 13 | -9.463 | 2.560 | 0.0002 |
| Crohn's disease | family | *Lactobacillaceae* | id.1836 | 72 | -0.031 | 0.015 | 0.037 |
|  | genus | *Eubacterium nodatum group* | id.11297 | 68 | 0.045 | 0.021 | 0.031 |
|  | genus | *Erysipelatoclostridium* | id.11381 | 72 | 0.037 | 0.012 | 0.002 |
|  | genus | *Holdemanella* | id.11393 | 72 | -0.029 | 0.014 | 0.045 |
|  | genus | *Lactobacillus* | id.1837 | 72 | -0.029 | 0.014 | 0.045 |
|  | genus | *Ruminiclostridium6* | id.11356 | 72 | -0.024 | 0.012 | 0.048 |
| Ulcerative colitis | genus | *Enterorhabdus* | id.820 | 9 | -14.655 | 6.782 | 0.031 |
|  | genus | *Flavonifractor* | id.2059 | 9 | -10.107 | 4.882 | 0.038 |
|  | genus | *Ruminococcus2* | id.11374 | 9 | -11.999 | 4.094 | 0.003 |

## Supplementary Table 11. Heterogeneity and pleiotropy test between IBD and GM.

| **Exposure** | **Outcome** | | | **Heterogeneity test** | | **Pleiotropy test** | |
| --- | --- | --- | --- | --- | --- | --- | --- |
|  | **Taxon level** | **Trait** | **MiBioGen ID** | **Q IVW** | ***P*** | **Egger intercept** | ***P*** |
| Inflammatory bowel disease | genus | *Eubacterium ruminantium group* | id.11340 | 4.946 | 0.960 | 0.008 | 0.648 |
|  | genus | *Barnesiella* | id.944 | 18.416 | 0.104 | -0.009 | 0.570 |
|  | genus | *Olsenella* | id.822 | 4.457 | 0.974 | 0.006 | 0.816 |
|  | genus | *Phascolarctobacterium* | id.2168 | 9.851 | 0.629 | 0.015 | 0.331 |
|  | genus | *Rikenellaceae rc9 gut group* | id.11191 | 7.188 | 0.784 | -0.043 | 0.292 |
|  | genus | *Ruminococcaceae UCG 003* | id.11361 | 3.793 | 0.987 | 0.004 | 0.780 |
|  | genus | *Ruminococcus2* | id.11374 | 6.811 | 0.870 | -0.015 | 0.240 |
| Crohn's disease | family | *Lactobacillaceae* | id.1836 | 78.975 | 0.242 | 0.007 | 0.289 |
|  | genus | *Eubacterium nodatum group* | id.11297 | 66.219 | 0.504 | -0.007 | 0.456 |
|  | genus | *Erysipelatoclostridium* | id.11381 | 74.195 | 0.374 | 0.004 | 0.543 |
|  | genus | *Holdemanella* | id.11393 | 81.713 | 0.181 | 0.003 | 0.632 |
|  | genus | *Lactobacillus* | id.1837 | 71.693 | 0.455 | 0.007 | 0.311 |
|  | genus | *Ruminiclostridium6* | id.11356 | 99.795 | 0.014 | 0.003 | 0.544 |
| Ulcerative colitis | genus | *Enterorhabdus* | id.820 | 10.947 | 0.205 | 0.059 | 0.257 |
|  | genus | *Flavonifractor* | id.2059 | 6.032 | 0.644 | -0.013 | 0.719 |
|  | genus | *Ruminococcus2* | id.11374 | 5.529 | 0.700 | -0.015 | 0.626 |

## Supplementary Table 12. Association between GM and biomarkers using inverse variance weighted method.

| **Taxon level** | **Trait** | **MiBioGen ID** | **SNPs** | **Beta** | **Standard error** | ***P*** |
| --- | --- | --- | --- | --- | --- | --- |
|  |  |  |  |  |  |  |
| **Outcome: C-reactive protein levels** | | | | | | |
| family | *Bacteroidaceae* | id.917 | 5 | 0.059 | 0.021 | 0.005 |
| family | *Bifidobacteriaceae* | id.433 | 6 | -0.061 | 0.020 | 0.003 |
| family | *Victivallaceae* | id.2255 | 9 | 0.013 | 0.007 | 0.046 |
| genus | *Actinomyces* | id.423 | 2 | -0.039 | 0.019 | 0.036 |
| genus | *Bacteroides* | id.918 | 5 | 0.059 | 0.021 | 0.005 |
| genus | *Bifidobacterium* | id.436 | 6 | -0.040 | 0.020 | 0.043 |
| genus | *Coprococcus1* | id.11301 | 7 | -0.036 | 0.014 | 0.010 |
| genus | *Dialister* | id.2183 | 2 | -0.055 | 0.023 | 0.019 |
| genus | *Eggerthella* | id.819 | 5 | 0.020 | 0.010 | 0.035 |
| genus | *Eubacterium brachy group* | id.11296 | 4 | 0.022 | 0.010 | 0.027 |
| genus | *Eubacterium oxidoreducens group* | id.11339 | 4 | 0.024 | 0.012 | 0.038 |
| genus | *Holdemania* | id.2157 | 6 | -0.026 | 0.011 | 0.025 |
| genus | *Howardella* | id.2000 | 7 | 0.014 | 0.007 | 0.046 |
| genus | *Odoribacter* | id.952 | 3 | -0.044 | 0.021 | 0.035 |
| genus | *Rikenellaceae RC9 gut group* | id.11191 | 6 | -0.019 | 0.007 | 0.011 |
| order | *Bifidobacteriales* | id.432 | 6 | -0.061 | 0.020 | 0.003 |
| order | *Burkholderiales* | id.2874 | 8 | 0.037 | 0.016 | 0.021 |
| **Outcome: Haemoglobin concentration** | | | | | | |
| class | *Erysipelotrichia* | id.2147 | 8 | -0.036 | 0.018 | 0.043 |
| family | *Erysipelotrichaceae* | id.2149 | 8 | -0.036 | 0.018 | 0.043 |
| family | *Family XIII* | id.1957 | 6 | -0.032 | 0.013 | 0.012 |
| family | *Lachnospiraceae* | id.1987 | 8 | 0.032 | 0.013 | 0.013 |
| genus | *Allisonella* | id.2174 | 4 | -0.025 | 0.007 | 0.001 |
| genus | *Clostridium innocuum group* | id.14397 | 2 | 0.036 | 0.017 | 0.028 |
| genus | *Eubacterium ventriosum group* | id.11341 | 8 | 0.027 | 0.011 | 0.013 |
| genus | *Lachnoclostridium* | id.11308 | 6 | -0.048 | 0.020 | 0.016 |
| genus | *Oscillospira* | id.2064 | 3 | -0.037 | 0.015 | 0.014 |
| genus | *Prevotella9* | id.11183 | 6 | -0.022 | 0.009 | 0.016 |
| genus | *Ruminococcaceae UCG002* | id.11360 | 10 | 0.037 | 0.011 | 0.001 |
| genus | *Sellimonas* | id.14369 | 6 | 0.015 | 0.007 | 0.028 |
| genus | *Turicibacter* | id.2162 | 6 | -0.037 | 0.012 | 0.002 |
| genus | *Tyzzerella3* | id.11335 | 9 | -0.025 | 0.011 | 0.030 |
| order | *Erysipelotrichales* | id.2148 | 8 | -0.036 | 0.018 | 0.043 |
| phylum | *Cyanobacteria* | id.1500 | 4 | 0.019 | 0.010 | 0.049 |
| phylum | *Verrucomicrobia* | id.3982 | 8 | -0.021 | 0.009 | 0.023 |
| **Outcome: Lymphocyte cell count** | | | | | | |
| family | *Lactobacillaceae* | id.1836 | 4 | 0.023 | 0.011 | 0.042 |
| family | *Ruminococcaceae* | id.2050 | 5 | -0.034 | 0.013 | 0.007 |
| genus | *Alloprevotella* | id.961 | 2 | 0.025 | 0.011 | 0.025 |
| genus | *Desulfovibrio* | id.3173 | 4 | 0.037 | 0.014 | 0.008 |
| genus | *Eubacterium brachy group* | id.11296 | 4 | 0.026 | 0.010 | 0.009 |
| genus | *Gordonibacter* | id.821 | 3 | -0.021 | 0.010 | 0.026 |
| genus | *Howardella* | id.2000 | 6 | -0.014 | 0.007 | 0.044 |
| genus | *Lachnospiraceae FCS020 group* | id.11314 | 7 | -0.036 | 0.018 | 0.043 |
| genus | *Ruminiclostridium9* | id.11357 | 4 | 0.082 | 0.025 | 0.001 |
| genus | *Ruminococcaceae UCG009* | id.11366 | 6 | -0.028 | 0.010 | 0.006 |
| genus | *Ruminococcus1* | id.11373 | 5 | 0.043 | 0.016 | 0.009 |
| genus | *Subdoligranulum* | id.2070 | 6 | -0.035 | 0.016 | 0.030 |
| phylum | *Bacteroidetes* | id.905 | 6 | 0.034 | 0.016 | 0.033 |
| phylum | *Firmicutes* | id.1672 | 6 | 0.057 | 0.023 | 0.013 |
| **Outcome: Neutrophill count** | | | | | | |
| family | *Family XI* | id.1936 | 7 | 0.036 | 0.014 | 0.014 |
| family | *Porphyromonadaceae* | id.943 | 3 | 0.059 | 0.025 | 0.019 |
| family | *Prevotellaceae* | id.960 | 9 | -0.029 | 0.012 | 0.016 |
| genus | *Anaerofilum* | id.2053 | 6 | -0.019 | 0.009 | 0.040 |
| genus | *Christensenellaceae R 7group* | id.11283 | 3 | -0.052 | 0.026 | 0.048 |
| genus | *Coprococcus2* | id.11302 | 3 | 0.050 | 0.019 | 0.010 |
| genus | *Lachnospiraceae UCG008* | id.11328 | 8 | -0.029 | 0.010 | 0.003 |
| genus | *Parabacteroides* | id.954 | 3 | 0.059 | 0.026 | 0.023 |
| genus | *Ruminococcaceae UCG013* | id.11370 | 7 | 0.029 | 0.014 | 0.041 |
| genus | *Ruminococcus2* | id.11374 | 7 | 0.028 | 0.013 | 0.038 |
| genus | *Subdoligranulum* | id.2070 | 7 | -0.039 | 0.017 | 0.024 |
| genus | *Terrisporobacter* | id.11348 | 2 | 0.044 | 0.021 | 0.041 |
| order | *Bacillales* | id.1674 | 4 | -0.031 | 0.010 | 0.001 |
| order | *Burkholderiales* | id.2874 | 8 | 0.049 | 0.018 | 0.005 |

## Supplementary Table 13. Heterogeneity and pleiotropy test between GM and biomarkers.

| **Taxon level** | **Trait** | **MiBioGen ID** | **Heterogeneity test** | | **Pleiotropy test** | |
| --- | --- | --- | --- | --- | --- | --- |
|  |  |  | **Q IVW** | ***P*** | **Egger intercept** | ***P*** |
| **Outcome: C-reactive protein levels** | | |  | |  |  |
| family | *Bacteroidaceae* | id.917 | 5.688 | 0.224 | 0.004 | 0.630 |
| family | *Bifidobacteriaceae* | id.433 | 11.594 | 0.041 | -0.006 | 0.237 |
| family | *Victivallaceae* | id.2255 | 9.835 | 0.277 | -0.003 | 0.585 |
| genus | *Actinomyces* | id.423 | 0.276 | 0.599 | 0.008 | 0.242 |
| genus | *Bacteroides* | id.918 | 5.688 | 0.224 | 0.004 | 0.630 |
| genus | *Bifidobacterium* | id.436 | 13.387 | 0.020 | -0.006 | 0.207 |
| genus | *Coprococcus1* | id.11301 | 2.338 | 0.886 | -0.003 | 0.352 |
| genus | *Dialister* | id.2183 | 1.071 | 0.301 | 0.002 | 0.356 |
| genus | *Eggerthella* | id.819 | 2.261 | 0.688 | 0.004 | 0.496 |
| genus | *Eubacterium brachy group* | id.11296 | 1.162 | 0.762 | 0.001 | 0.840 |
| genus | *Eubacterium oxidoreducens group* | id.11339 | 3.220 | 0.359 | 0.001 | 0.871 |
| genus | *Holdemania* | id.2157 | 1.363 | 0.928 | 0.002 | 0.728 |
| genus | *Howardella* | id.2000 | 5.086 | 0.533 | -0.002 | 0.646 |
| genus | *Odoribacter* | id.952 | 0.876 | 0.645 | -0.005 | 0.521 |
| genus | *Rikenellaceae RC9 gut group* | id.11191 | 1.465 | 0.917 | -0.007 | 0.327 |
| order | *Bifidobacteriales* | id.432 | 11.594 | 0.041 | -0.006 | 0.237 |
| order | *Burkholderiales* | id.2874 | 9.236 | 0.236 | 0.004 | 0.287 |
| **Outcome: Haemoglobin concentration** | | |  | |  |  |
| class | *Erysipelotrichia* | id.2147 | 16.593 | 0.020 | -0.008 | 0.123 |
| family | *Erysipelotrichaceae* | id.2149 | 16.593 | 0.020 | -0.008 | 0.123 |
| family | *Family XIII* | id.1957 | 0.605 | 0.988 | 0.001 | 0.891 |
| family | *Lachnospiraceae* | id.1987 | 8.539 | 0.287 | 0.002 | 0.562 |
| genus | *Allisonella* | id.2174 | 1.939 | 0.585 | -0.005 | 0.509 |
| genus | *Clostridium innocuum group* | id.14397 | 2.000 | 0.157 | 0.001 | 0.690 |
| genus | *Eubacterium ventriosum group* | id.11341 | 4.781 | 0.687 | -0.002 | 0.601 |
| genus | *Lachnoclostridium* | id.11308 | 10.420 | 0.064 | -0.013 | 0.038 |
| genus | *Oscillospira* | id.2064 | 1.809 | 0.405 | -0.007 | 0.549 |
| genus | *Prevotella9* | id.11183 | 2.908 | 0.714 | 0.002 | 0.544 |
| genus | *Ruminococcaceae UCG002* | id.11360 | 12.790 | 0.172 | 0.002 | 0.454 |
| genus | *Sellimonas* | id.14369 | 7.140 | 0.210 | 0.009 | 0.092 |
| genus | *Turicibacter* | id.2162 | 7.421 | 0.191 | -0.009 | 0.280 |
| genus | *Tyzzerella3* | id.11335 | 29.626 | 0.001 | -0.010 | 0.329 |
| order | *Erysipelotrichales* | id.2148 | 16.593 | 0.020 | -0.008 | 0.123 |
| phylum | *Cyanobacteria* | id.1500 | 1.871 | 0.600 | 0.003 | 0.672 |
| phylum | *Verrucomicrobia* | id.3982 | 1.739 | 0.973 | -0.002 | 0.493 |
| **Outcome: Lymphocyte cell count** | | |  | |  |  |
| family | *Lactobacillaceae* | id.1836 | 1.251 | 0.741 | 0.004 | 0.428 |
| family | *Ruminococcaceae* | id.2050 | 3.682 | 0.451 | -0.005 | 0.453 |
| genus | *Alloprevotella* | id.961 | 1.034 | 0.309 | 0.004 | 0.745 |
| genus | *Desulfovibrio* | id.3173 | 0.723 | 0.868 | 0.003 | 0.745 |
| genus | *Eubacterium brachy group* | id.11296 | 3.451 | 0.327 | -0.005 | 0.344 |
| genus | *Gordonibacter* | id.821 | 0.761 | 0.684 | 0.007 | 0.564 |
| genus | *Howardella* | id.2000 | 3.406 | 0.638 | 0.003 | 0.435 |
| genus | *Lachnospiraceae FCS020 group* | id.11314 | 18.160 | 0.006 | -0.007 | 0.017 |
| genus | *Ruminiclostridium9* | id.11357 | 5.476 | 0.140 | 0.002 | 0.853 |
| genus | *Ruminococcaceae UCG009* | id.11366 | 4.513 | 0.478 | 0.010 | 0.162 |
| genus | *Ruminococcus1* | id.11373 | 3.112 | 0.539 | 0.001 | 0.980 |
| genus | *Subdoligranulum* | id.2070 | 6.281 | 0.280 | -0.005 | 0.178 |
| phylum | *Bacteroidetes* | id.905 | 5.384 | 0.371 | 0.004 | 0.239 |
| phylum | *Firmicutes* | id.1672 | 10.408 | 0.064 | 0.003 | 0.687 |
| **Outcome: Neutrophill count** | | |  | |  |  |
| family | *Family XI* | id.1936 | 27.216 | 0.001 | -0.022 | 0.070 |
| family | *Porphyromonadaceae* | id.943 | 0.880 | 0.644 | -0.012 | 0.564 |
| family | *Prevotellaceae* | id.960 | 8.353 | 0.400 | -0.004 | 0.156 |
| genus | *Anaerofilum* | id.2053 | 1.743 | 0.883 | 0.001 | 0.811 |
| genus | *Christensenellaceae R 7group* | id.11283 | 2.245 | 0.325 | 0.002 | 0.873 |
| genus | *Coprococcus2* | id.11302 | 0.919 | 0.632 | 0.014 | 0.529 |
| genus | *Lachnospiraceae UCG008* | id.11328 | 8.068 | 0.327 | 0.011 | 0.191 |
| genus | *Parabacteroides* | id.954 | 2.263 | 0.323 | 0.004 | 0.863 |
| genus | *Ruminococcaceae UCG013* | id.11370 | 6.340 | 0.386 | 0.002 | 0.652 |
| genus | *Ruminococcus2* | id.11374 | 4.192 | 0.651 | 0.003 | 0.363 |
| genus | *Subdoligranulum* | id.2070 | 7.885 | 0.247 | 0.003 | 0.409 |
| genus | *Terrisporobacter* | id.11348 | 1.749 | 0.186 | 0.002 | 0.160 |
| order | *Bacillales* | id.1674 | 3.226 | 0.358 | 0.002 | 0.837 |
| order | *Burkholderiales* | id.2874 | 10.090 | 0.184 | 0.003 | 0.586 |

## Supplementary Table 14. Baseline characteristics of IBD participants by incident CRC in UK Biobank.

| **Characteristic^1^** | **Control (N=4,893)** | **Incident CRC (N=98)** | ***P*-value^2^** |
| --- | --- | --- | --- |
| **Age** **at** **baseline (years), mean (SD)** | 56.9 (8.0) | 59.4 (7.5) | <0.001 |
| **Sex, n (%)** |  |  | 0.093 |
| Female | 2,516 (51.4) | 42 (42.9) |  |
| Male | 2,377 (48.6) | 56 (57.1) |  |
| **Ethnic, n (%)** |  |  |  |
| White | 4,664 (95.3) | 94 (96.0) |  |
| Asian | 117 (2.4) | 1 (1.0) |  |
| African | 37 (0.8) | 0 (0.0) |  |
| Mixed background | 16 (0.3) | 1 (1.0) |  |
| Unknown | 59 (1.2) | 2 (2.0) |  |
| **Education, n (%)** |  |  | 0.500 |
| College/university degree | 3,850 (78.7) | 77 (78.4) |  |
| Non-college/university degree | 992 (20.3) | 19 (19.6) |  |
| Unknown | 51 (1.0) | 2 (2.0) |  |
| **Townsend deprivation index^3^, mean (SD)** | -1.25 (3.09) | -1.25 (3.12) | 0.834 |
| **BMI (kg/m2), n (%)** |  |  | 0.214 |
| 18.5-24.9 | 1,103 (22.5) | 21 (21.4) |  |
| 24.9-29.9 | 2,115 (43.2) | 50 (51.0) |  |
| >29.9 | 1,618 (33.1) | 25 (25.5) |  |
| Unknown | 57 (1.2) | 2 (2.1) |  |
| **Smoking status, n (%)** |  |  | 0.279 |
| Never | 474 (9.7) | 5 (5.1) |  |
| Previous | 2,085 (42.6) | 42 (42.9) |  |
| Current | 2,314 (47.3) | 51 (52.0) |  |
| Unknown | 20 (0.4) | 0 (0.0) |  |
| **Family CRC history of parents and siblings^4^, n (%)** |  |  | 0.939 |
| Yes | 463 (9.5) | 10 (10.2) |  |
| No | 3,991 (81.6) | 80 (81.6) |  |
| Unknown | 439 (9.0) | 8 (8.2) |  |
| **CRC screening history^5^, n (%)** |  |  | 0.244 |
| Yes | 627 (12.8) | 9 (9.2) |  |
| No | 76 (1.6) | 0 (0.0) |  |
| Unknown | 4,190 (85.6) | 89 (90.8) |  |
| **WCRF/AICR HLI score^6^, mean (SD)** | 3.0 (0.9) | 3.0 (0.8) | 0.831 |
| **CRP (mg/L), mean (SD)** | 3.5 (4.7) | 3.5 (4.0) | 0.996 |
| **Platelet (10^9/L), mean (SD)** | 265.6 (63.9) | 271.4 (66.5) | 0.375 |
| **Hgb (g/dL), mean (SD)** | 14.0 (1.2) | 13.9 (1.1) | 0.679 |
| **Albumin (g/L), mean (SD)** | 44.5 (2.8) | 43.8 (2.6) | 0.008 |
| **NLR, mean (SD)** | 2.7 (1.3) | 2.8 (1.2) | 0.721 |
| **PLR, mean (SD)** | 160.8 (65.6) | 167.9 (74.8) | 0.293 |
| **SII (10^9/L), mean (SD)** | 734.6 (418.3) | 761.4 (392.9) | 0.53 |
| **Inflammatory score, n (%)** |  |  | 0.077 |
| Low | 495 (10.1) | 4 (4.1) |  |
| Low-moderate | 1,232 (25.2) | 19 (19.4) |  |
| Moderate-high | 1,543 (31.5) | 40 (40.8) |  |
| High | 1,090 (22.3) | 21 (21.4) |  |

^1^ Mean (SD) values and n (%) are reported for continuous and categorical variables, respectively.

^2^ t test and Pearson's Chi-squared test are tested for continuous and categorical variables, respectively.

^3^ The Townsend index is a measure of material deprivation within a population. The measure incorporates four variables: Unemployment (as a percentage of those aged 16 and over who are economically active), Non-car ownership (as a percentage of all households), Non-home ownership (as a percentage of all households), and Household overcrowding.

^4^ The illnesses of parents were collected from participants who indicated they were not adopted as a child, and who indicated whether their natural father/mother was still alive or had died. The illnesses of siblings were collected from participants who indicated they were not adopted as a child, and that they had at least one natural brother or sister.

^5^ CRC screening history was defined based on the answer of assessment center touchscreen question "Have you ever had a screening test for bowel (colorectal) cancer? (Please include tests for blood in the stool/faeces or a colonoscopy or a sigmoidoscopy)".

^6^ HLI included diet, alcohol consumption, smoking, physical activity, BMI, and waist circumference, specific items definition and corresponding score allocations are detailed in the Supplementary Table 2.

Abbreviations: BMI, body mass index, CRC, colorectal cancer, HLI, healthy lifestyle index, WCRF/AICR: the World Cancer Research Fund/American Institute for Cancer Research.

## Supplementary Table 15. Associations between disease severity associated laboratory biomarker score and risk of CRC in IBD, UC, CD patients.

| Score category | Person years | n CRC/N total (% CRC) | Model 1 HR (95%CIs)^1^ | *P-value* | Model 2 HR (95%CIs)^2^ | *P-value* |
| --- | --- | --- | --- | --- | --- | --- |
| IBD patients |  |  |  |  |  |  |
| Low | 10092.93 | 9/871 (1.03%) | Ref |  | Ref |  |
| Low-moderate | 12502.74 | 23/1088 (2.11%) | 2.10 (0.97,4.55) | 0.059 | 2.39 (1.07,5.35) | 0.034 |
| Moderate-high | 24727.45 | 45/2151 (2.09%) | 2.16 (1.05,4.42) | 0.036 | 2.48 (1.17,5.29) | 0.018 |
| High | 10105.79 | 21/881 (2.38%) | 2.63 (1.20,5.77) | 0.016 | 3.07 (1.35,7.00) | 0.008 |
| UC patients |  |  |  |  |  |  |
| Low | 7450.27 | 6/640 (0.09%) | Ref |  | Ref |  |
| Low-moderate | 8682.58 | 18/757 (2.38%) | 2.62 (1.04,6.62) | 0.041 | 2.62 (1.04,6.63) | 0.041 |
| Moderate-high | 16122.05 | 35/1399 (2.50%) | 2.85 (1.20,6.80) | 0.018 | 2.91 (1.22,6.95) | 0.016 |
| High | 5824.92 | 14/505 (2.77%) | 3.36 (1.28,8.81) | 0.014 | 3.45 (1.31,9.09) | 0.012 |
| CD patients |  |  |  |  |  |  |
| Low | 2642.65 | 3/231 (1.30%) | Ref |  | Ref |  |
| Low-moderate | 3820.16 | 5/331 (1.51%) | 1.18 (0.28,4.95) | 0.82 | 1.79 (0.35,9.30) | 0.487 |
| Moderate-high | 8605.4 | 10/752 (1.33%) | 1.08 (0.29,3.92) | 0.912 | 1.54 (0.33,7.07) | 0.58 |
| High | 4280.88 | 7/376 (1.86%) | 1.60 (0.41,6.25) | 0.496 | 2.52 (0.52,12.30) | 0.254 |

^1^ Model 1: Estimated from the Cox regression model with age as the underlying time scale, adjusted for sex, age at recruitment, race (white, Asian, African, mixed background, unknown) in UK Biobank.

^2^ Model 2: Further adjusted for Townsend deprivation index (continuous), region of the recruitment assessment center, educational level (college/university degree, non-college/university degree, unknown), CRC screening history (no, yes, unknown), family history of CRC (no, yes, unknown), standard polygenic risk score for CRC (continuous), healthy lifestyle index and IBD disease course in UK Biobank.

Abbreviations: CI, confidence interval, HR, hazard ratio, CRC, colorectal cancer, IBD, inflammatory bowel disease, UC, ulcerative colitis, CD, Crohn's disease.

## Supplementary Table 16. Associations between disease severity associated laboratory biomarker score and risk of CRC in IBD, UC, CD patients by sex.

| Score category | Person years | n CRC/N total (% CRC) | Model 1 HR (95%CIs)^1^ | *P-value* | Model 2 HR (95%CIs)^2^ | *P-value* |
| --- | --- | --- | --- | --- | --- | --- |
| **Male** |  |  |  |  |  |  |
| IBD |  |  |  |  |  |  |
| Low | 6301.96 | 6/544 (1.10%) | Ref |  | Ref |  |
| Low-moderate | 6360.88 | 14/553 (2.53%) | 2.24 (0.86,5.83) | 0.099 | 2.79 (1.00,7.74) | 0.05 |
| Moderate-high | 11342.74 | 24/1004 (2.39%) | 2.19 (0.89,5.35) | 0.087 | 2.74 (1.04,7.20) | 0.041 |
| High | 3650.12 | 12/332 (3.61%) | 3.35 (1.26,8.94) | 0.016 | 4.25 (1.49,12.10) | 0.007 |
| UC |  |  |  |  |  |  |
| Low | 4812.74 | 4/414 (0.97%) | Ref |  | Ref |  |
| Low-moderate | 4512.85 | 11/394 (2.79%) | 2.84 (0.90,8.91) | 0.075 | 2.91 (0.93,9.16) | 0.068 |
| Moderate-high | 7604.59 | 18/674 (2.67%) | 2.82 (0.95,8.34) | 0.061 | 2.92 (0.99,8.65) | 0.053 |
| High | 2145.62 | 9/194 (4.64%) | 4.95 (1.52,16.08) | 0.008 | 5.23 (1.60,17.08) | 0.006 |
| CD |  |  |  |  |  |  |
| Low | 1489.22 | 2/130 (1.54%) | Ref |  | Ref |  |
| Low-moderate | 1848.03 | 3/159 (1.89%) | 1.18 (0.20,7.08) | 0.855 | 2.80 (0.28,27.97) | 0.381 |
| Moderate-high | 3738.15 | 6/330 (1.82%) | 1.18 (0.24,5.84) | 0.841 | 2.45 (0.29,20.84) | 0.412 |
| High | 1504.5 | 3/138 (2.17%) | 1.42 (0.24,8.48) | 0.704 | 3.33 (0.34,32.99) | 0.304 |
| **Female** |  |  |  |  |  |  |
| IBD |  |  |  |  |  |  |
| Low | 3790.97 | 3/327 (0.92%) | Ref |  | Ref |  |
| Low-moderate | 6141.87 | 9/535 (1.68%) | 1.87 (0.51,6.90) | 0.348 | 1.84 (0.50,6.82) | 0.36 |
| Moderate-high | 13384.72 | 21/1147 (1.83%) | 2.00 (0.60,6.69) | 0.263 | 1.99 (0.59,6.71) | 0.264 |
| High | 6455.68 | 9/549 (1.64%) | 1.89 (0.51,6.98) | 0.341 | 1.75 (0.47,6.55) | 0.405 |
| UC |  |  |  |  |  |  |
| Low | 2637.53 | 2/226 (0.88%) | Ref |  | Ref |  |
| Low-moderate | 4169.73 | 7/363 (1.93%) | 2.20 (0.46,10.58) | 0.326 | 2.04 (0.42,9.86) | 0.375 |
| Moderate-high | 8517.46 | 17/725 (2.34%) | 2.64 (0.61,11.41) | 0.195 | 2.55 (0.59,11.08) | 0.211 |
| High | 3679.30 | 5/311 (1.61%) | 1.88 (0.36,9.70) | 0.451 | 1.60 (0.30,8.38) | 0.58 |
| CD |  |  |  |  |  |  |
| Low | 1153.43 | 1/101 (0.99%) | Ref |  | Ref |  |
| Low-moderate | 1972.13 | 2/172 (1.16%) | 1.28 (0.12,14.14) | 0.841 | 1.18 (0.11,13.29) | 0.891 |
| Moderate-high | 4867.26 | 4/422 (0.95%) | 0.99 (0.11,8.84) | 0.991 | 0.87 (0.10,7.91) | 0.905 |
| High | 2776.38 | 4/238 (1.68%) | 1.85 (0.21,16.66) | 0.582 | 1.70 (0.18,15.75) | 0.641 |

^1^ Model 1: Estimated from the Cox regression model with age as the underlying time scale, adjusted for sex, age at recruitment, race (white, Asian, African, mixed background, unknown) in UK Biobank.

^2^ Model 2: Further adjusted for Townsend deprivation index (continuous), region of the recruitment assessment center, educational level (college/university degree, non-college/university degree, unknown), CRC screening history (no, yes, unknown), family history of CRC (no, yes, unknown), standard polygenic risk score for CRC (continuous), healthy lifestyle index and IBD disease course in UK Biobank.

Abbreviations: CI, confidence interval, HR, hazard ratio, CRC, colorectal cancer, IBD, inflammatory bowel disease, UC, ulcerative colitis, CD, Crohn's disease.

## Supplementary Table 17. Sensitivity analysis of the associations between disease severity associated laboratory biomarker score and risk of CRC in IBD, UC, CD patients excluding dual diagnosis.

| Score category | Person years | n CRC/N total (% CRC) | Model 1 HR (95%CIs)^1^ | *P-value* | Model 2 HR (95%CIs)^2^ | *P-value* |
| --- | --- | --- | --- | --- | --- | --- |
| IBD patients |  |  |  |  |  |  |
| Low | 8902.34 | 7/767 (0.91%) | Ref |  | Ref |  |
| Low-moderate | 10846.24 | 20/947 (2.11%) | 2.42 (1.02,5.73) | 0.045 | 2.47 (1.04,5.85) | 0.040 |
| Moderate-high | 21442.98 | 36/1865 (1.93%) | 2.29 (1.02,5.17) | 0.045 | 2.36 (1.04,5.32) | 0.039 |
| High | 7994.3 | 16/697 (2.30%) | 2.92 (1.20,7.15) | 0.019 | 3.04 (1.24,7.46) | 0.016 |
| UC patients |  |  |  |  |  |  |
| Low | 6995.49 | 6/600 (1.00%) | Ref |  | Ref |  |
| Low-moderate | 7922.89 | 16/692 (2.31%) | 2.43 (0.95,6.23) | 0.064 | 2.43 (0.95,6.24) | 0.064 |
| Moderate-high | 14788.84 | 30/1282 (2.34%) | 2.54 (1.05,6.12) | 0.038 | 2.57 (1.06,6.21) | 0.036 |
| High | 4828.49 | 13/420 (3.10%) | 3.59 (1.35,9.52) | 0.010 | 3.66 (1.37,9.78) | 0.010 |
| CD patients |  |  |  |  |  |  |
| Low | 1906.85 | 1/167 (0.60%) | Ref |  | Ref |  |
| Low-moderate | 2923.36 | 4/255 (1.57%) | 2.67 (0.30,23.95) | 0.380 | 2.68 (0.30,24.14) | 0.378 |
| Moderate-high | 6654.14 | 6/583 (1.03%) | 1.78 (0.21,14.83) | 0.595 | 1.72 (0.20,14.35) | 0.619 |
| High | 3165.81 | 3/277 (1.08%) | 1.96 (0.20,18.99) | 0.561 | 2.07 (0.21,20.12) | 0.531 |

^1^ Model 1: Estimated from the Cox regression model with age as the underlying time scale, adjusted for sex, age at recruitment, race (white, Asian, African, mixed background, unknown) in UK Biobank.

^2^ Model 2: Further adjusted for Townsend deprivation index (continuous), region of the recruitment assessment center, educational level (college/university degree, non-college/university degree, unknown), CRC screening history (no, yes, unknown), family history of CRC (no, yes, unknown), standard polygenic risk score for CRC (continuous), healthy lifestyle index and IBD disease course in UK Biobank.

Abbreviations: CI, confidence interval, HR, hazard ratio, CRC, colorectal cancer, IBD, inflammatory bowel disease, UC, ulcerative colitis, CD, Crohn's disease.

**Supplementary Figures Legends**

Supplementary Figure 1. Flowchart of the participants excluded from the cohort study.

Supplementary Figure 2. Association between UC, CD and CRC outcomes based on two step MR analysis.


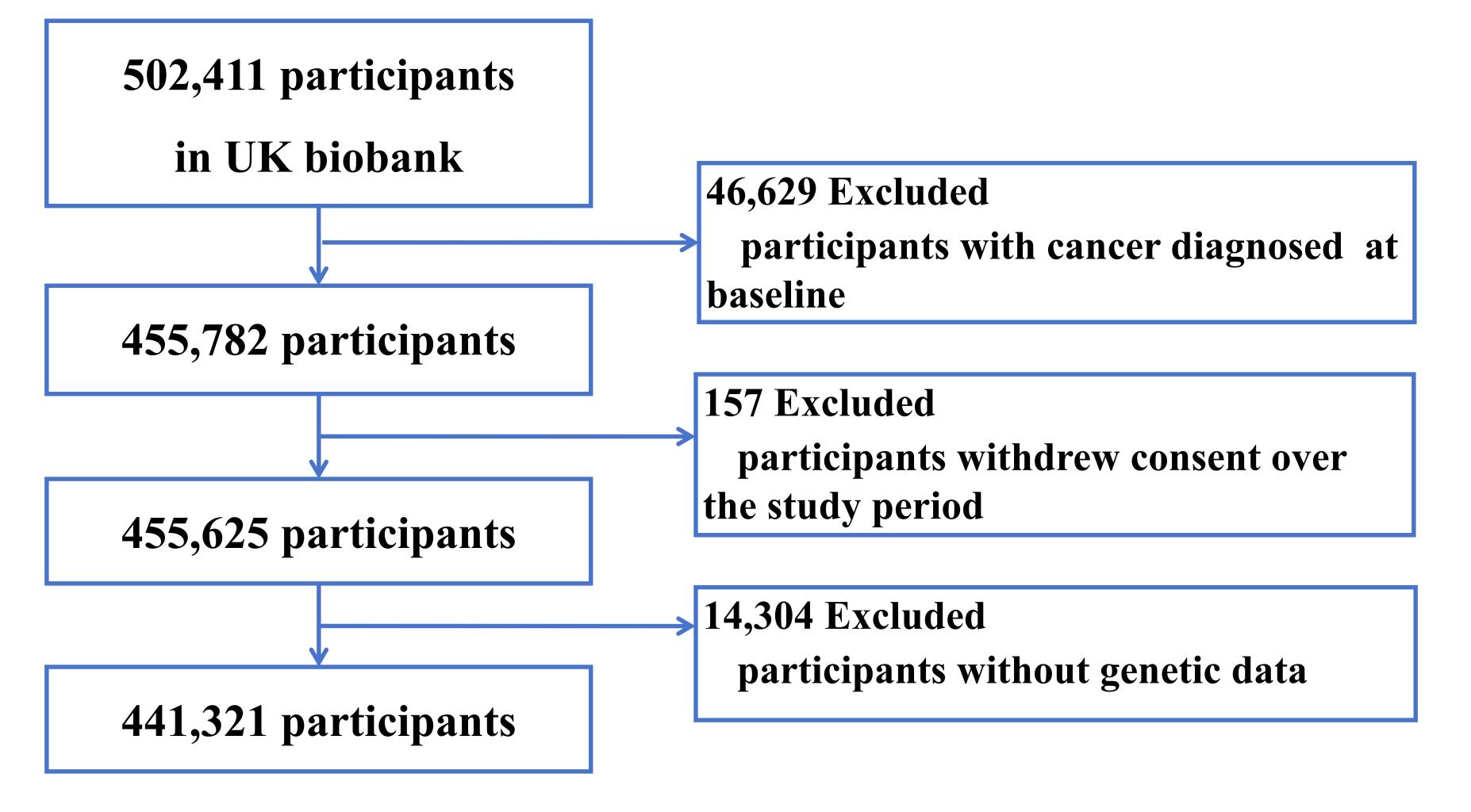


## Supplementary Figure 1. Flowchart of the participants excluded from the cohort study


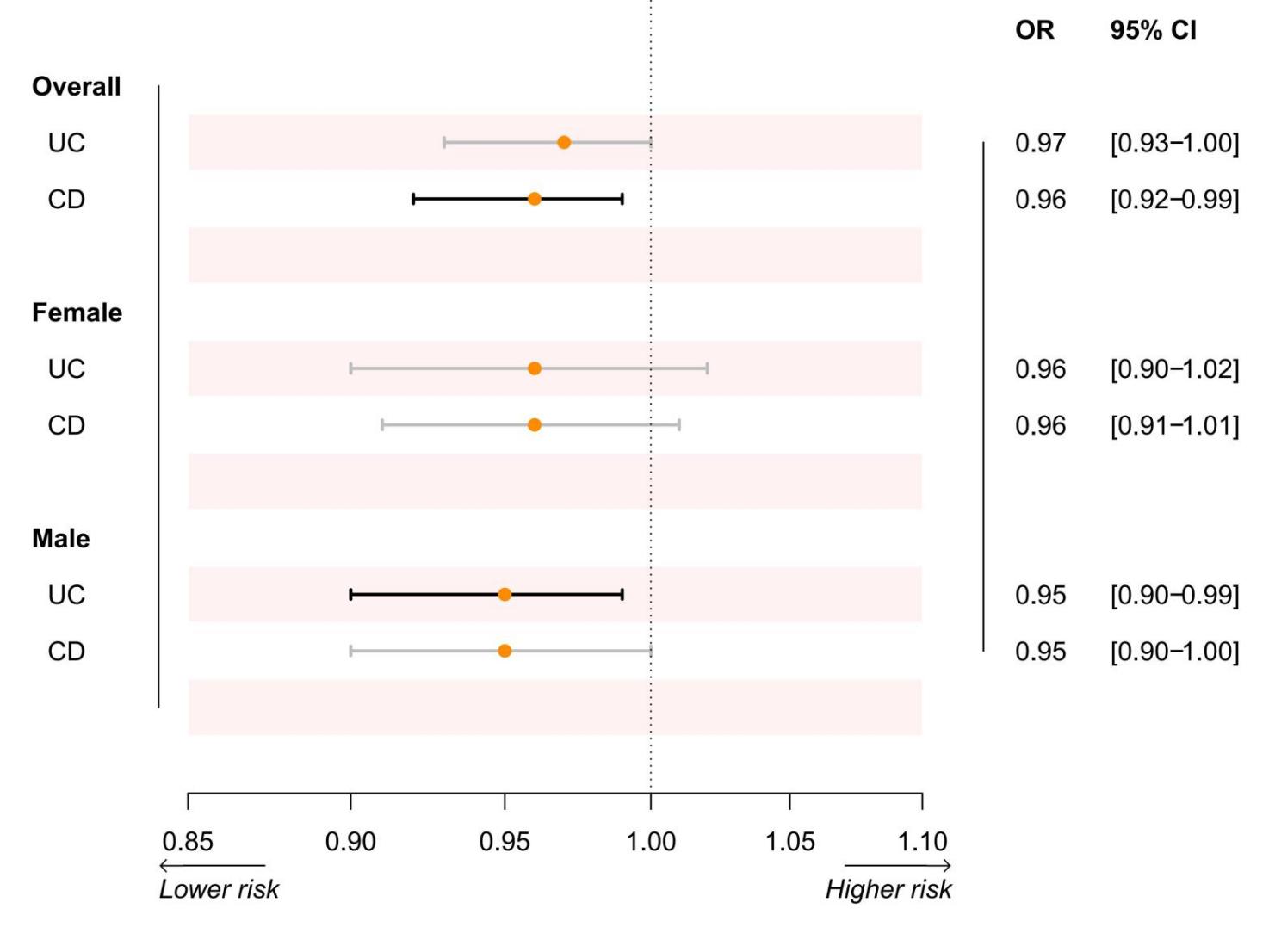


## Supplementary Figure 2. Association between UC, CD and CRC outcomes based on two step MR analysis.

Abbreviations: CRC, colorectal cancer, IBD, inflammatory bowel disease, UC, ulcerative colitis, CD, Crohn's disease.
